# Metabolic reprogramming in PGPR reveals cross-feeding-driven physiological shifts and metabolic adaptations

**DOI:** 10.3389/fmicb.2025.1668025

**Published:** 2025-11-24

**Authors:** Kamogelo Mmotla, Farhahna Allie, Thendo Mafuna, Manamele D. Mashabela, Msizi I. Mhlongo

**Affiliations:** 1 Faculty of Science, Department of Biochemistry, University of Johannesburg, Johannesburg, South Africa; 2 Imbewu Metabolomics Research Group, Faculty of Science, Department of Biochemistry, University of Johannesburg, Auckland Park Kingsway Campus, Johannesburg, South Africa; 3 Faculty of Science, Research Centre for Plant Metabolomics, University of Johannesburg, South Africa

**Keywords:** *Priestia megaterium*, *Bacillus licheniformis*, cross-feeding, plant growth-promoting rhizobacteria, metabolomics, SDGs

## Abstract

**Introduction:**

Microbial interactions in the rhizosphere are fundamental to soil health, plant growth, and ecosystem stability. Among these interactions, metabolic cross-feeding, the exchange of metabolites between microorganisms, plays a critical role in shaping microbial community structure and function. This study investigates the metabolic interplay between two PGPR (*Priestia megaterium and Bacillus licheniformis*), focusing on how metabolite exchange influences bacterial growth and metabolic reprogramming.

**Methods:**

An integrative metabolomics approach was employed to examine metabolic exchanges between *P. megaterium* and *B. licheniformis*. Cultures were grown individually and in co-culture, followed by extraction of extracellular metabolites at distinct growth phases. Metabolomic profiling was conducted using ultra-performance liquid chromatography-mass spectrometry (UPLC-MS). Data preprocessing and feature extraction were followed by molecular networking and multivariate statistical analysis to identify discriminant metabolites. Pathway enrichment and functional annotation were performed using KEGG and MetaboAnalyst to pinpoint key metabolic pathways altered during cross-feeding interactions.

**Results and discussion:**

Metabolomic analysis revealed distinct metabolic shifts driven by reciprocal metabolite exchange between *P. megaterium* and *B. licheniformis*. Metabolites secreted by *B. licheniformis* exhibited a growth-inhibitory effect on *P. megaterium*, while those from *P. megaterium* stimulated the growth of *B. licheniformis*. Multivariate data analysis demonstrated significant variation in the production of amino acids, fatty acids, and cyclic lipopeptides across growth phases. Pathway enrichment identified the phenylalanine, tyrosine, and tryptophan biosynthesis (PTTB) pathway as a central metabolic hub mediating these interactions. The regulation of aromatic amino acid metabolism appeared critical in determining whether interactions were cooperative or competitive. The observed metabolic reprogramming reflects adaptive strategies employed by PGPR to thrive under nutrient-limited conditions, balancing cooperation and competition through selective metabolite secretion. These findings offer systems-level insight into the mechanistic basis of cross-feeding and highlight the potential of integrating metabolomics to guide microbial consortia design for agricultural applications. Understanding these metabolic determinants supports the development of tailored biofertilizer formulations that enhance soil fertility and plant resilience.

**Conclusion:**

This study demonstrates that metabolite cross-feeding induces distinct metabolic reprogramming between *Priestia megaterium* and *Bacillus licheniformis*, underpinning adaptive interactions in nutrient-limited environments. These findings provide a mechanistic basis for microbial consortia design and biofertilizer optimization. Future multi-omics and systems-level investigations should elucidate the genetic and regulatory determinants of these metabolic exchanges, advancing sustainable biotechnological innovations aligned with SDGs 9, 12, and 13.

## Introduction

Microbial activity in the soil is centered in the plant rhizosphere, the small region of soil surrounding plant roots (De la Fuente Cantó et al., 2020). It is a focal point for microorganisms, characterized by intense and complex interactions between plants and microbes (Prasad et al., 2019). Within the ecosystem, various microbes, including bacteria, fungi, protozoa, algae, and other soil microbes, coexist and interact constantly with one another. However, studies show that bacteria that foster plant growth and development are notably more prevalent than other microbes in the rhizosphere (Bahadur et al., 2016; Kalam et al., 2017). These microorganisms are commonly known as Plant Growth-Promoting Rhizobacteria (PGPR) and play a critical role in plant growth and development by performing various functions such as nitrogen fixation, phosphate solubilization, induction of systemic resistance, heavy metal reduction and phytohormone production (including auxins, gibberellins, cytokinins, etc.), decomposition of crop residue and suppression of phytopathogens, among other essential activities. Notably, species such as *Priestia megaterium* and *Bacillus licheniformis* are well-studied examples of PGPR that make meaningful contributions to improving soil fertility and enhancing plant health (Biedendieck et al., 2021). Additionally, PGPR have been reported to confer resistance and tolerance to biotic and abiotic stresses in plants, essential traits, particularly in agriculture (Etesami and Maheshwari, 2018; He et al., 2019; Mhlongo et al., 2020; Mashabela et al., 2023). The functional characteristics of PGPR typically result from various interactions among themselves, using metabolic activities to form intricate networks of cooperation within their microbial community. Through these activities, PGPR can alter their chemical environment by engaging in metabolite exchanges, depending on other PGPR to obtain metabolites. This, in turn, fosters the emergence of new ecological interactions. This metabolite exchange is commonly known as cross-feeding and encompasses a diverse array of metabolites, ranging from organic acids to vitamins, and most commonly, volatile organic compounds (VOCs) (Mataigne et al., 2021; Lopez and Wingreen, 2022). Microbial cross-feeding refers to the collaboration between microorganisms, where metabolites produced through the metabolism of one microorganism (the donor) are utilized and undergo further metabolism by another microorganism (the receiver) (Mataigne et al., 2021). Cross-feeding, therefore, significantly influences the composition and function of microbial communities and is a key factor in promoting microbial diversity, thereby enabling numerous species to coexist in natural environments.

Cross-feeding can be classified as either unidirectional, where one microorganism benefits from another, or bidirectional, where both microorganisms derive advantages from each other's secretions (D'Souza et al., 2018; Mataigne et al., 2021). This categorization is based on the directionality of the exchange or the specific compounds involved in the interaction. As an illustration, cross-feeding occurs between *Rhodococus ruber* and *Bacillus cereus*. Where *R. ruber* breaks down tetrahydrofuran, yielding acidic metabolites that *B. cereus* utilizes. In turn, *B. cereus* regulates pH levels and secretes micronutrients crucial for *R. ruber's* growth (Liu et al., 2019). Another example involves two mutants of *Pseudomonas stuzeri*. Depending on the pH conditions, these mutants exhibit a transition from competing to engaging in significant mutual support by exchanging nitrite, a substance known for its toxicity under acidic conditions. This shift illustrates how microorganisms can transition from unidirectional to bidirectional interactions, influenced by environmental factors such as pH (Borer et al., 2020). However, microbial cross-feeding may extend beyond interactions between pairs of microorganisms, as multiple recipient species could gain advantages from the metabolites produced by a single provider species (Mataigne et al., 2021). Although cross-feeding appears to be a common phenomenon in nature, certain aspects remain unclear, particularly how it influences the temporal dynamics of primary and secondary metabolite production and the subsequent effects on microbial adaptation. Environmental conditions, such as nutrient limitations, play a crucial role in driving cross-feeding interactions by altering microbial metabolism. Under such conditions, bacteria release metabolic by-products, including amino acids, organic acids, and secondary metabolites, which can be utilized by neighboring microbes to support growth and survival (Ilyas et al., 2017). These metabolic exchanges create a dynamic interaction that influences not only individual bacterial fitness but also the stability of the community. For example, Pande et al. (2013) demonstrated that metabolic cross-feeding can enhance microbial growth by dividing metabolic labor. In their study, genetically modified *Escherichia coli* strains, unable to synthesize certain amino acids but capable of excreting others, were co-cultured to observe their metabolic interactions. Surprisingly, these strains exhibited higher growth rates than wild-type *E. coli*, suggesting that the accumulation and exchange of metabolites provided a selective advantage (Pande et al., 2013).

The investigation of metabolic composition using metabolomics has provided a fresh perspective on various biological/biochemical processes, particularly in understanding how microorganisms interact through metabolic exchange (Brunetti et al., 2018). This has enabled the identification of key mediator metabolites involved in organism interactions, leading to a better understanding of regulatory pathways. Hence, in this study, we applied metabolomics to monitor metabolic changes during PGPR cross-feeding, revealing alterations in primary and secondary metabolism. This method provided insights into how PGPR enhance resource efficiency, modulates bioactive metabolite synthesis, shedding light on the metabolic pathways and regulatory networks involved in facilitating cooperative behavior among PGPR populations in the rhizosphere.

## Methodology

### Bacterial strains, growth conditions and isolation

The bacterial strains used in this study were *P. megaterium* (PM) and *B. licheniformis* (BL). *P. megaterium* was obtained from the University of Johannesburg, South Africa, and maintained as a glycerol stock at −80°C. Meanwhile, *B. licheniformis* was isolated from microbial inoculant (Efficient Microbes Pro-soil, Westville KZN, RSA).

To isolate *B. licheniformis*, a series of dilutions was performed by diluting 1 ml of microbial inoculum in LB Broth. The dilutions ranged from 10^−1^ to 10^−5^ and were subsequently plated on agar media containing (Meat extract, Peptone, Sodium chloride, and Yeast extract). The colonies that developed on the agar media were subsequently sent to Inqaba Biotech (Inqaba Biotechnical Industries Pty Ltd) for 16S rDNA sequencing. The sequencing procedure carried out by Inqaba involved extracting genomic DNA from the cultures using the Quick-DNATM Fungal/Bacterial Miniprep Kit (Zymo Research, Catalog No. D6005). For 16S rDNA gene amplification, the forward primer 27f (5-AGAGTTTGATCMTGGCTCAG-3) and the reverse primer 1492r (5-CGGTTACCTTGTTACGACTT-3) were used. The general PCR protocol was executed with the following settings: an initial cycle of 94 °C for 5 min, followed by 35 cycles of 94 °C for 30 s, 50 °C for 30 s, and 68 °C for 1 min. The final extension was performed at 68 °C for 10 min. The integrity of the PCR amplicons was assessed by visualizing them on a 1% agarose gel (CSL-AG500, Cleaver Scientific Ltd) stained with EZ-vision^®^ Bluelight DNA Dye at a concentration of 5 μL per 50 mL of gel. The gels were incubated at room temperature for 10 min before visualization. The NEB Fast Ladder (N3238) was utilized as a size standard on all gels. The amplicons were purified for sequencing (Zymo Research, ZR-96 DNA Sequencing Clean-up KitTM, Catalog No. D4050) and sequenced in the forward and reverse direction (Nimagen, BrilliantDyeTM Terminator Cycle Sequencing Kit V3.1, BRD3-100/1000) using the ABI 3730xl Genetic Analyser (Applied Biosystems, Thermo Fisher Scientific). For data Analysis FinchTV (https://finchtv.software.informer.com/1.4/) was used to view the raw chromatogram files (.abi). The CLC Bio Main Workbench was used to assemble the forward and reverse sequencing reads to form a consensus sequence for each sample. BLASTn analysis (with default parameters) was performed on the NCBI website (https://blast.ncbi.nlm.nih.gov/Blast.cgi) to determine if a sequence in the database matches the query sequence above a certain threshold (99% query coverage; 99% identity).

### Experimental design for microbial cross-feeding

#### M9 Liquid media preparation

The amount of 1L of M9 media composition was prepared by weighing and mixing salts (2.56 % Na_2_HPO_4_ (Merck (Pty) Ltd, S.A), 0.6% KH_2_PO_4_ (Merck KGaA, Germany), 0.1% NaCl (RLS chemicals, S.A), 0.2% NH_4_Cl (Merck KGaA, Germany), 0.05% MgSO_4_ (Merck KGaA, Germany). The salts were autoclaved and cooled at room temperature. Then 0.1% of glucose (Acechem, S.A), 0.2% of malic acid (Merck KGaA, Italy), 0.05% MgSO_4_ (Merck KGaA, Italy) and 0.002% of 1 M CaCl (Sigma-Aldrich, Germany) were filtered through a 0.22 μl nylon syringe filter and added into the autoclaved salts.

#### *P. megaterium* and *B. licheniformis* cross-feeding

The metabolic exchange (ME) between *P. megaterium* and *B. licheniformis* was studied in three independent biological replicates. Both treated (cross-feed strains) and control (single cultures of the strains) sets were included in the experiments. Initially, *P. megaterium* and *B. licheniformis* were cultured in 50 mL M9 media and allowed to incubate overnight at 32 °C with thermal precision of ±2 °C. The optical density (OD) at 600 nm was continuously monitored until it reached an OD of 0.1. Subsequently, the cultures underwent centrifugation at 4,700 rpm for 15 min at room temperature using a fixed-angle rotor. The resulting supernatant was carefully filtered through a 0.22 μm nylon syringe filter (GVS, USA) into a sterile 50 mL Erlenmeyer flask. For the ME-1 experiment, 0.1 OD of *P. megaterium* (recipient strain) was introduced into 50 mL of M9 media containing extracts from *B. licheniformis* (donor strain). The cultures were then incubated overnight at 30 °C, with OD measurements for every 6 h, recorded at 600 nm using a spectrophotometer. Post-incubation, the cultures were transferred into new 50 mL tubes and centrifuged at 4,700 rpm for 15 min at room temperature using a fixed-angle rotor. The supernatant was then filtered into new 15 mL Falcon tubes, and the pellet was stored at −80 °C. This procedure was repeated at 6-h intervals, spanning from 0 h to 36 h, as delineated in Supplementary Tables 1, 2. The identical experimental protocol was also repeated for ME-2 (*B. licheniformis* as recipient - *P. megaterium* as donor).

### Metabolites re-constitution

The frozen samples underwent a freeze-drying process lasting 72 h. Following this, 50% methanol (Romil, Cambridge, UK) and 0.1% formic acid (Sigma-Aldrich, USA) solutions were prepared, and 3,000 μl of these solutions were added to each 15 mL Falcon tube containing the dried samples. Subsequently, the samples were homogenized using a rotor-stator homogeniser at a rpm of 17,500 for 20 s, and the resulting mixture was then filtered through 0.22 μm nylon syringe filters into 2 mL vials for subsequent metabolomics analysis. The reconstituted samples were kept at −20 °C before analysis to preserve metabolic stability, in accordance with the recommendations outlined by Pinu and Villas-Boas (2017). Under *Storage of Extracellular Microbial Samples*.

#### Ultra-performance liquid chromatography-mass spectrometry (UPLC-MS) analysis

Data acquisition was based on the method described by Jäger et al. (2021), with minor modifications. Chromatographic separation was performed using an ACQUITYTM PREMIER HSS T3 1.8 μm Column (Waters). The mobile phases consist of (A) water containing 0.05% formic acid and (B) acetonitrile with 0.05% formic acid. The gradient elution was performed at a flow rate of 0.4 mL/min and a column temperature of 40 °C, starting from 1% to 99% B over 21 min, then held at 100% B for 1.5 min, and finally returned to 1% B for an additional 1.5 min. The injection volume was 3 μL. High-resolution mass spectrometry was used in positive electrospray ionization (ESI) mode, employing MSE acquisition, which collects data independently of precursor ion selection by alternating between low and high collision energies, covering a mass range of 100–1,500 Da. The instrument parameters included a capillary voltage of +2,800 V, a cone voltage of +30 V, a source temperature of 120 °C, a desolvation temperature of 450 °C, a desolvation gas flow of 600 L/h, and a cone gas flow of 50 L/h. Data were acquired in centroid mode at an approximate resolution of 10,000 with a scan time of 0.1 s, ensuring over 10 data points per chromatographic peak. Internal mass calibration was performed using the Lockspray interface (Waters) by infusing a leucine-enkephalin solution (500 ng/mL) at a flow rate of 15 μL/min. Data acquisition and instrument control were managed using MassLynx 4.1 software (Waters).

#### Data processing and multivariate data analysis

Both centroid ESI positive and negative raw data from Ultra-High Performance Liquid Chromatography with Quadrupole Time-of-Flight Mass Spectrometry UPLC-Q-TOF-MS analysis were examined. Data visualization and processing were carried out using MassLynx XSTM 4.1 software (Waters Connect, Manchester, UK). The UNIFY application manager of the MassLynx software was utilized for data pre-processing, generating a data matrix consisting of retention time (Rt)-m/z variable pairs, with m/z peak intensities for each sample. The MS data were processed using the following parameters: an Rt range of 0.67-16.05, a mass range of 100–1,500 Da, a mass tolerance of 0.05 Da and a noise elimination threshold of 10. The data were then normalized using MassLynx software (Waters Connect, Manchester, UK).

The cleaned data matrices, which included a complex covariance structure, were imported into MetaboAnalyst (a web-based platform dedicated to comprehensive metabolomics) software version 6.0 (https://www.metaboanalyst.ca/home.xhtml) for data analysis. The study initially employed unsupervised machine learning techniques, such as principal component analysis (PCA), to effectively reduce the data's dimensionality while preserving its structures and characteristics, including natural groupings, trends, outliers and potential sub-structures (Tugizimana et al., 2013). After this unsupervised modeling, the insights gained were used to apply supervised machine learning methods, specifically orthogonal partial least squares discriminant analysis (OPLS-DA). This projection-based algorithm facilitates sample classification in a low-dimensional space, enabling the identification and selection of metabolite features that distinguish between different groups or classes.

### Molecular networking in global natural product social platform (GNPS)

Mass spectral data files acquired in ESI positive mode (.raw format, Waters) were first collected and then converted into the analysis base file (ABF) format to enable compatibility with MS-DIAL software (version 4.90) (Tsugawa et al., 2015), using the Reifycs ABF converter (https://www.reifycs.com/AbfConverter/). This conversion process facilitated streamlined data processing and analysis within the MS-DIAL platform. The ABF files were subsequently processed and deconvoluted for data-independent acquisition (DIA), using specific parameters: a mass accuracy of 0.5 Da for both MS1 and MS2, a minimum peak height threshold of 10 amplitude, a peak detection slice width of 0.1 Da, a sigma value of 0.5, and a retention tolerance of 0.2 min. After processing, 8,814 and 536 peak features were identified for *P. megaterium* (control and treated MCF-1) and *B. licheniformis* (control and treated MCF-2). The GNPS2 export files, including the MGF file and the Sample Table (feature quantification), were then uploaded to GNPS2 (https://gnps2.org/homepage) for molecular network generation. Feature-based molecular networks (FBMN) were then constructed using a precursor and fragment ion mass tolerance of 0.5 Da across both treated and control samples of each species, with a minimum cosine score of 0.7 and a minimum of 4 matched fragment ions. The networks were visualized using Cytoscape software (https://cytoscape.org/), a powerful tool for network analysis and visualization. Within Cystoscape, distinct classes of metabolites were visualized and organized to facilitate a clear interpretation and analysis of the relationships and groupings present in the data.

### Metabolite annotation

A combination of semi/-automated and manual methods was used to annotate metabolites. The first step was automated annotation in MS-DIAL using MSP spectral libraries for metabolomics, which included all publicly available MS/MS libraries. Additional annotation was accomplished by using FBMN to search GNPS libraries. A thorough manual procedure was used to enhance and validate these annotations. This process involved determining the molecular formula for specific m/z and retention time features, linking the molecular formula with databases such as PubChem (https://pubchem.ncbi.nlm.nih.gov/), MassBank (https://massbank.eu/MassBank/), and KEGG (https://www.genome.jp/kegg), and verifying structural accuracy by examining fragmentation patterns and comparing the results with previously reported metabolites. This method enabled the annotation of metabolites based on MSI levels 2 and 3.

## Results

### Unexpected inhibitory effect of *B. licheniformis* metabolites on *P. megaterium* growth over time

**Figure 1:**
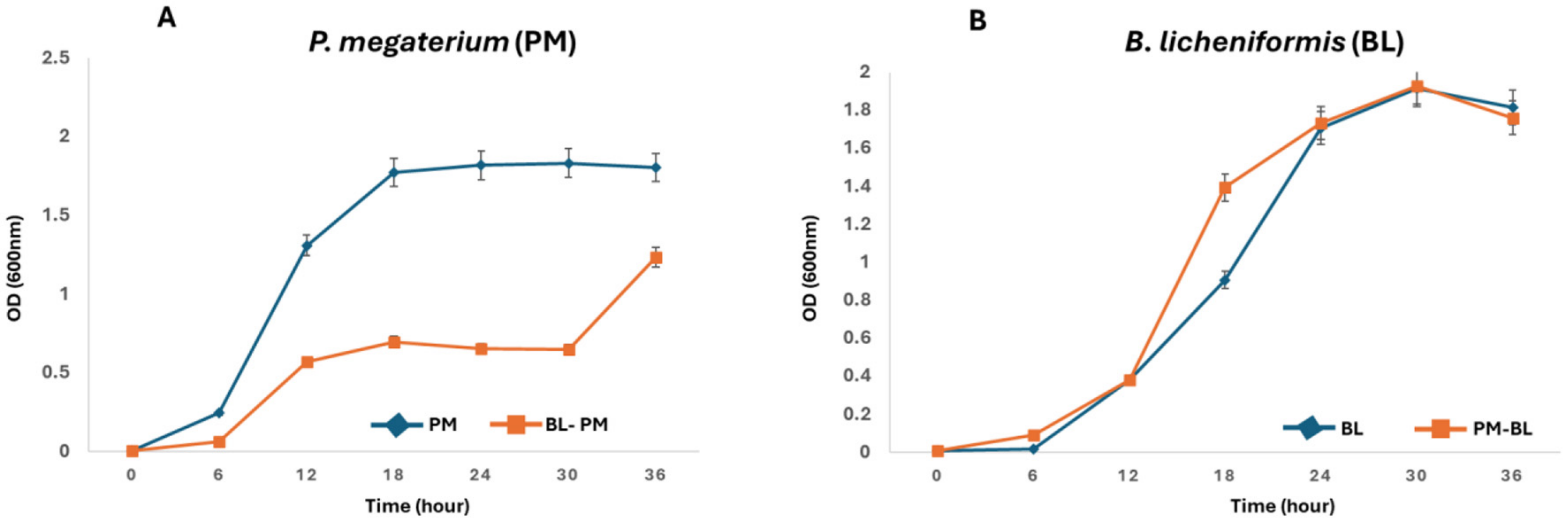
Reciprocal growth curves of *P. megaterium* and *B. licheniformis* as a receiver and donor strains: **(A)** Growth of *P. megaterium* (receiver) was monitored in monoculture (PM) and when cultured in the metabolites produced by *B. licheniformis* (donor, PM-BL). **(B)** Conversely, the growth of *B. licheniformis* (receiver) was assessed in monoculture (BL) and when cultured in metabolites produced by *P. megaterium* (donor, BL-PM). Optical density at 600 nm (OD600) was measured at 6-h intervals over a 36-hour period. PM-BL and BL-PM indicate receiver strains grown in the metabolites of the donor, while PM and BL denote monoculture controls. Data represent the mean ± standard error from the triplicate cultures.

The growth curves depicted in Figure 1A reveal unexpected inhibitory effects of *B. licheniformis* metabolites on the growth of *P. megaterium* over a 36-h period. Contrary to our hypothesis, where we anticipated that *B. licheniformis* metabolites would enhance *P. megaterium* growth, the results showed a significant decrease in *P. megaterium's* optical density when cross-fed with *B. licheniformis* metabolites compared to *P. megaterium* grown in monoculture. Throughout the course, *P. megaterium* exhibited lower OD values in the presence of *B. licheniformis* metabolites, indicating that these metabolites either suppressed or were not essential for *P. megaterium's* growth. This surprising outcome suggests that metabolites produced by *B. licheniformis* may contain inhibitory compounds that negatively affect *P. megaterium's* proliferation, highlighting the complexity of microbial interactions and the potential for antagonistic relationships within the PGPR community. On the other hand, the metabolic makeup of *B. licheniformis* may not consist of essential metabolites and nutrients required for the growth and proliferation of *P. megaterium*.

Contrary to ME-1, the metabolites produced by *P. megaterium* had a positive effect on *B. licheniformis* growth (Figure 1B). In the monoculture condition, *B. licheniformis* exhibited typical growth kinetics, gradually increasing in OD as expected. However, when *B. licheniformis* was cultured in the presence of *P. megaterium*, there was a notable enhancement in growth, characterized by a higher OD reading (12–18 h) compared to monocultured *B. licheniformis*. These results suggest that *P. megaterium* secretes metabolites that promote the proliferation of *B. licheniformis*, possibly through the provision of nutrients, growth factors, or other beneficial interactions.

### Multivariate data analysis: metabolic profiling of PGPR-induced shifts resulting from cross-feeding interactions

**Figure 2:**
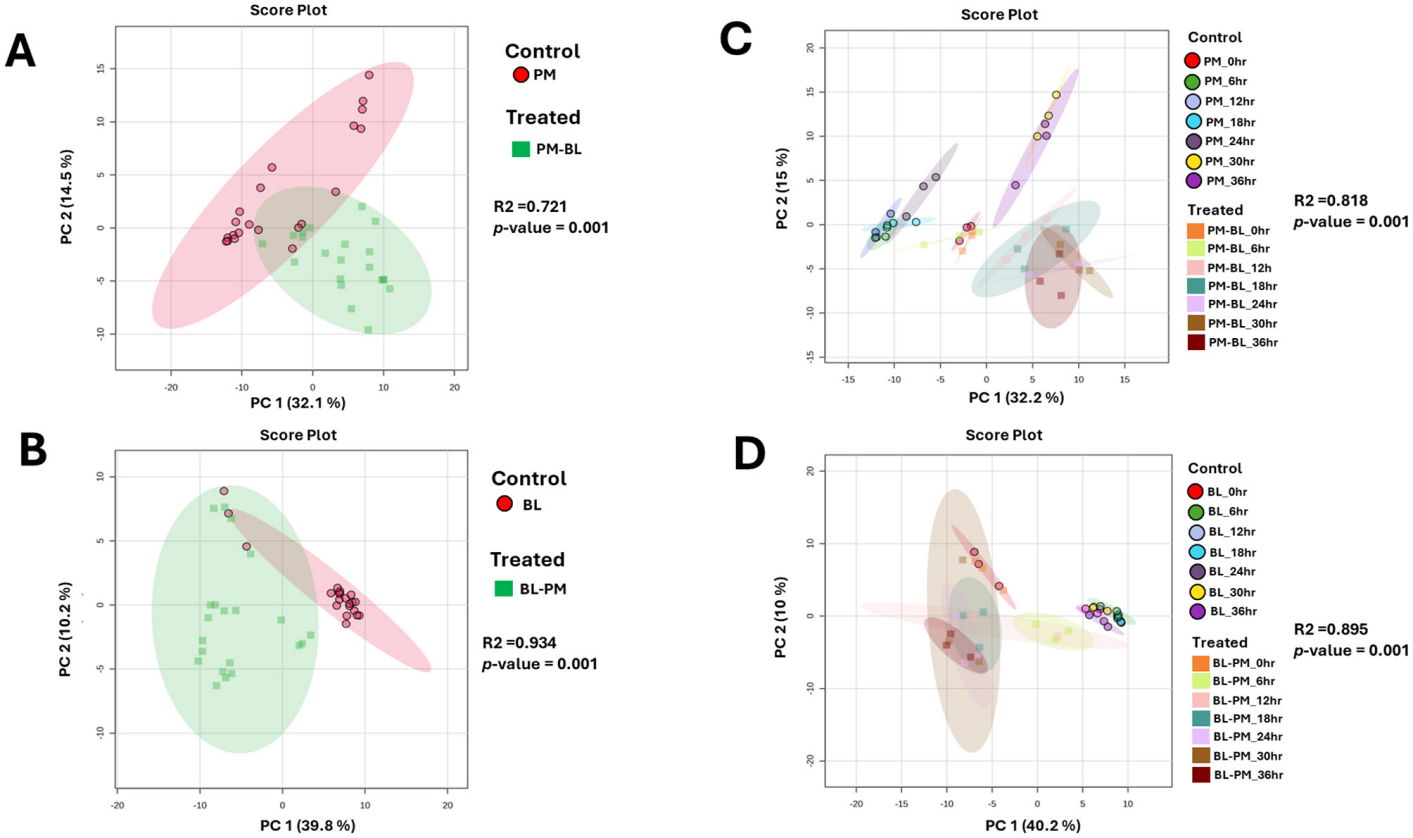
Unsupervised statistical analysis of *P. megaterium* and *B. licheniformis* samples collected in positive ESI mode. **(A, C)** Display the overall metabolic profiles of mono-cultured (red) and treated (green) *P. megaterium* and *B. licheniformis*. **(B, D)** highlight the time-dependent variations and changes in metabolic profiles observed in **(A, C)**. The data projected above were median-normalized, log-transformed and *Pareto*-scaled in MetaboAnalyst for correlation and predictability scores of *R*2 = 0.72065 and *p-*value = 0.001 **(A)**; R2 = 0.93415 and *p-*value of 0.001 **(B)**, R2 = 0.81814 and *p-*value = 0.001 **(C)**, R2 = 0.89477 and *p-*value = 0.001 **(D)**.

We analyzed the time-dependent exo-metabolomes of *P. megaterium* treated with *B. licheniformis* culture extracts (ME-1) and *B. licheniformis* treated with *P. megaterium* culture extracts (ME-2). The overall metabolic profiles of both species in control and treated samples were overlapping, indicating that certain metabolites are shared between the treated (ME) and control samples (Figures 2A, B). Such a pattern suggests that the core metabolic processes of both species remained largely stable despite the treatment, possibly reflecting a level of metabolic resilience or adaptation. The overlap was partially expected, given that both species share fundamental metabolic pathways that may not be easily disrupted. However, time-point principal component analysis (PCA) provided deeper insights into the dynamic metabolic changes over time. For *P. megaterium*, PC1 explained 32.2% of total variance, representing the major metabolic differences induced by treatment, while PC2 accounted for 15%, capturing additional variation linked to subtle shifts in metabolite abundance and composition (Figure 2C). Similarly, for *B. licheniformis*, PC1 and PC2 explained 40.2% and 10% of the variance, respectively (Figure 2D). These percentages indicate that a substantial portion of the metabolic variability can be attributed to the experimental conditions and time-dependent metabolic reprogramming.

At the initial 0-h time point, control and treated samples clustered closely, reflecting similar metabolic states before exposure to the reciprocal culture extracts. As the incubation progressed, microbial metabolism of the introduced extracts led to the production of new metabolites, degradation of certain compounds, and shifts in metabolite concentrations. This metabolic reprogramming drove the divergence of treated samples from controls, resulting in distinct clustering patterns over time. Specifically, treated *P. megaterium* cultures showed separation from controls during the later growth phases (30–36 h), while treated *B. licheniformis* displayed distinct clustering from controls during both early (6–12 h) and later (18–36 h) growth stages.

Aligned with the PCA results, the Hierarchical Cluster Analysis (HiCA plot) in Supplementary Figure 1 enhances our understanding of the chemical relationships and separations between the control and treated groups across various time points. The distinct clustering in HiCA closely mirrors the distribution of these groups observed in the PCA, with control samples, at certain hours, displaying the greatest divergence from treated (ME) at similar time points. This separation suggests that control and treated samples represent metabolically distinct profiles that may shift dynamically over time, potentially shaped by strain-specific characteristics. This clustering pattern is further supported by a descriptive heatmap in Figure 7, which highlights key metabolites linked to these interactions at various time points.

### GNPS profiling of metabolic alterations in the receiver bacterium triggered by the exposure to the donor's metabolites

**Figure 3:**
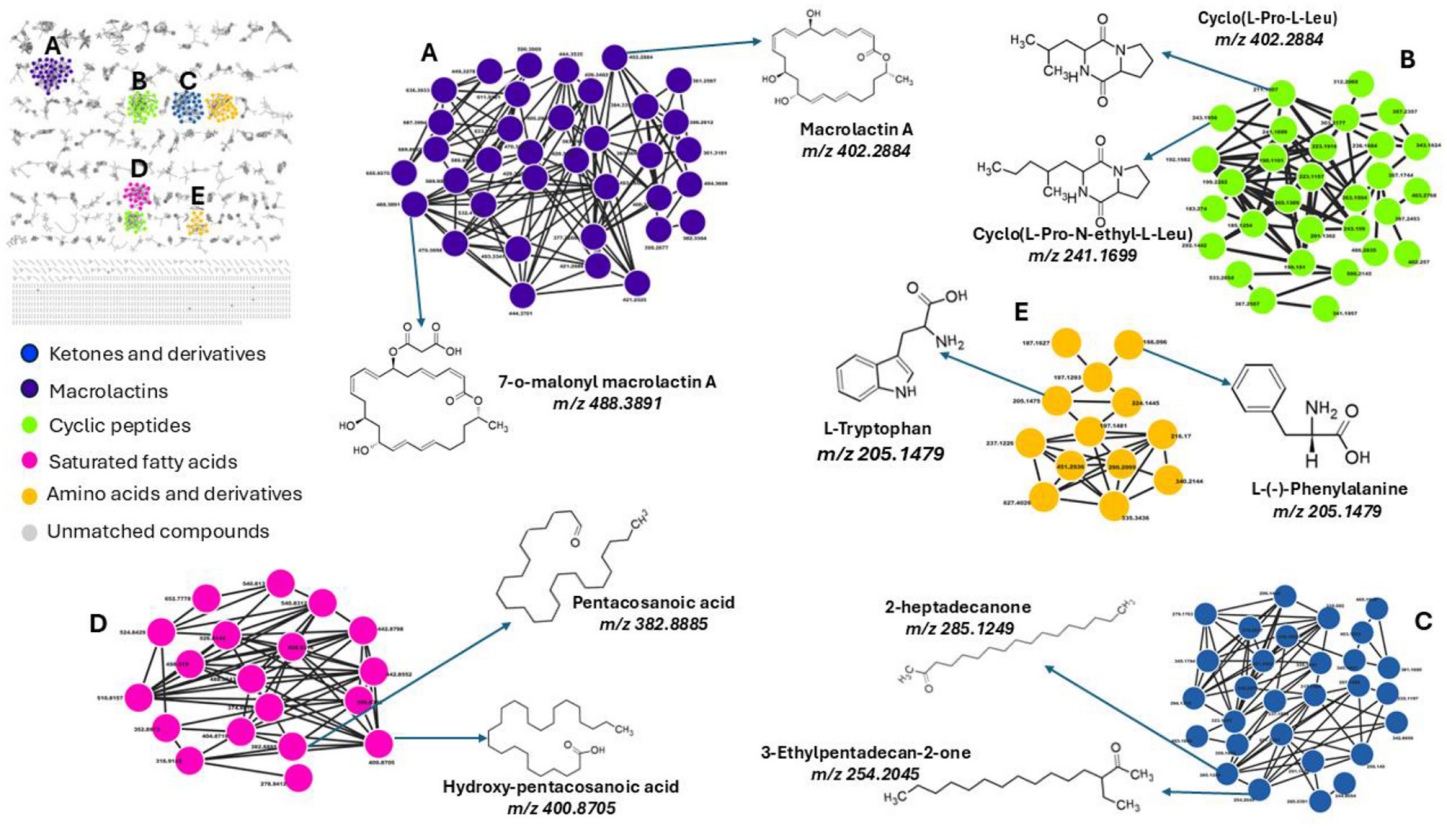
Molecular network of metabolites produced by bacteria, categorized by chemical classes. The molecular network shows metabolites grouped into clusters representing different chemical classes: **(A)** Macrolactins (purple), **(B)** Cyclic peptides (green), **(C)** Ketones and derivatives (blue), **(D)** Saturated fatty acids (pink), and **(E)** Amino acids and derivatives (yellow). Unmatched compounds are indicated in gray. Representative structures from each cluster are highlighted, including macrolactin A (m/z 402.2884), cyclo(L-Pro-N-ethyl-L-Leu) (m/z 241.1699), 2-heptadecanone (m/z 285.1249), pentacosanoic acid (m/z 382.8885), and L-tryptophan (m/z 205.1479). Each cluster illustrates structurally related compounds, connected by edges representing spectral similarity.

To facilitate metabolite annotation and identification, molecular networking (MN) was employed to group experimental spectra into molecular families based on spectral similarities. When connected to related or comparable annotated metabolites, this method facilitates the identification of molecular families that contain unknown metabolites. Unlike classic molecular networking, FBMN incorporates quantitative and MS1 chromatographic data, including peak intensities, retention time and ion mobility separation. This additional data facilitates further statistical analysis and annotation tools, enabling the accurate identification of isomers with comparable MS2 spectra within molecular networks. This study's analysis of the bacterial exo-metabolome included both identified and unidentified classes of metabolites with no matches (Figure 3). Each node in a cluster represents a corresponding chemically related entity, which is connected to other nodes by edges.

**Figure 4:**
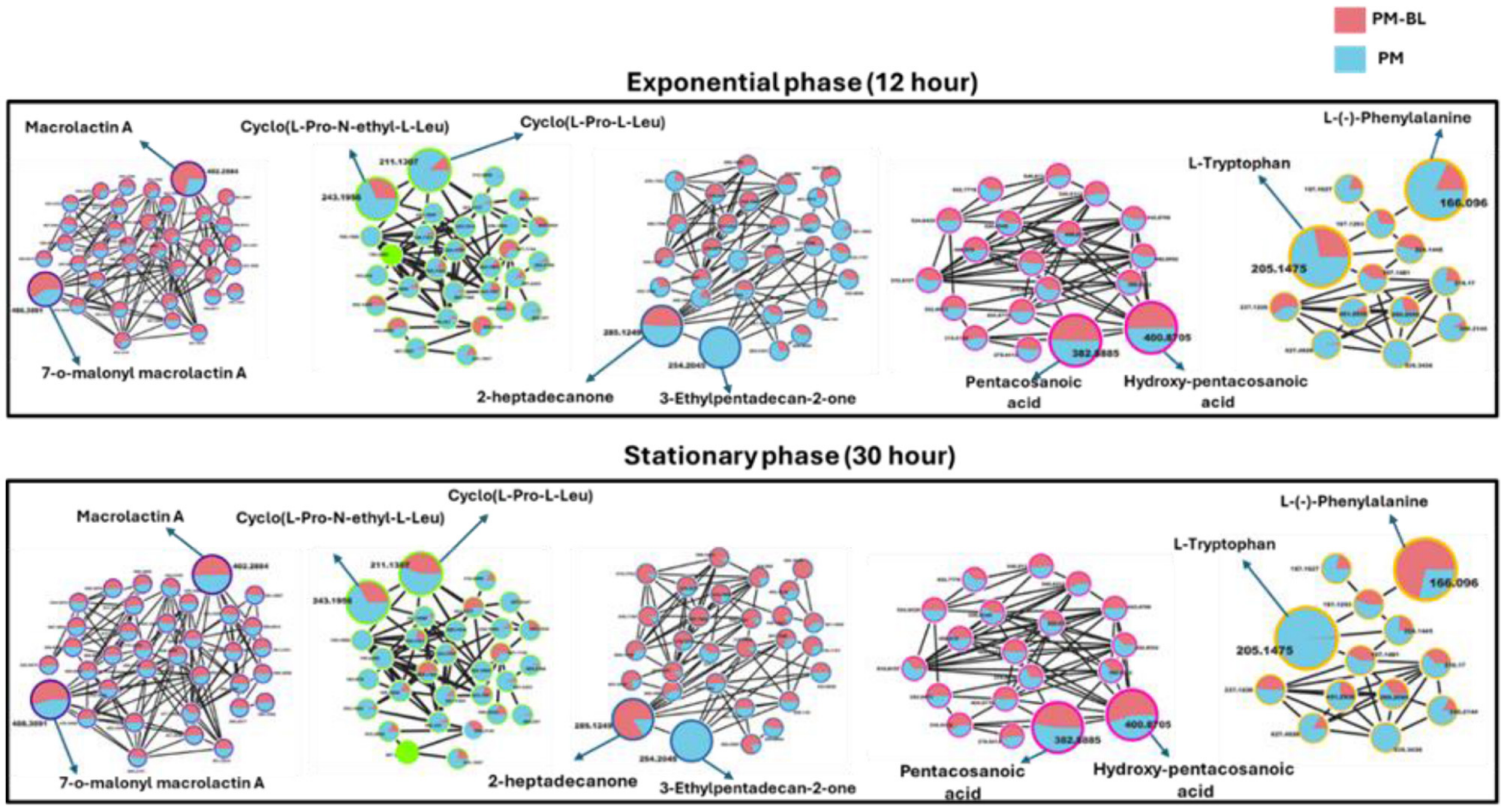
Comparative molecular network of metabolites produced by *P. megaterium* (PM) and treated with *B. licheniformis* (PM-BL) at exponential and stationary phases. The molecular networks illustrate metabolites detected at 12 h (Exponential phase) and 30 h (Stationary phase) for PM (blue nodes) and PM-BL (pink nodes). Node size indicates whether a metabolite was annotated (larger nodes) or unannotated (smaller nodes), while the edges represent spectral similarity. Representative metabolites in each cluster are labeled, including macrolactin A, cyclo(L-Pro-N-ethyl-L-Leu), 2-heptadecanone, pentacosanoic acid, hydroxy-pentacosanoic acid, L-tryptophan, and L-phenylalanine. Metabolite profiles reveal shifts between monoculture (PM) and cross-fed conditions (PM-BL), with variations in abundance and cluster size.

**Figure 5:**
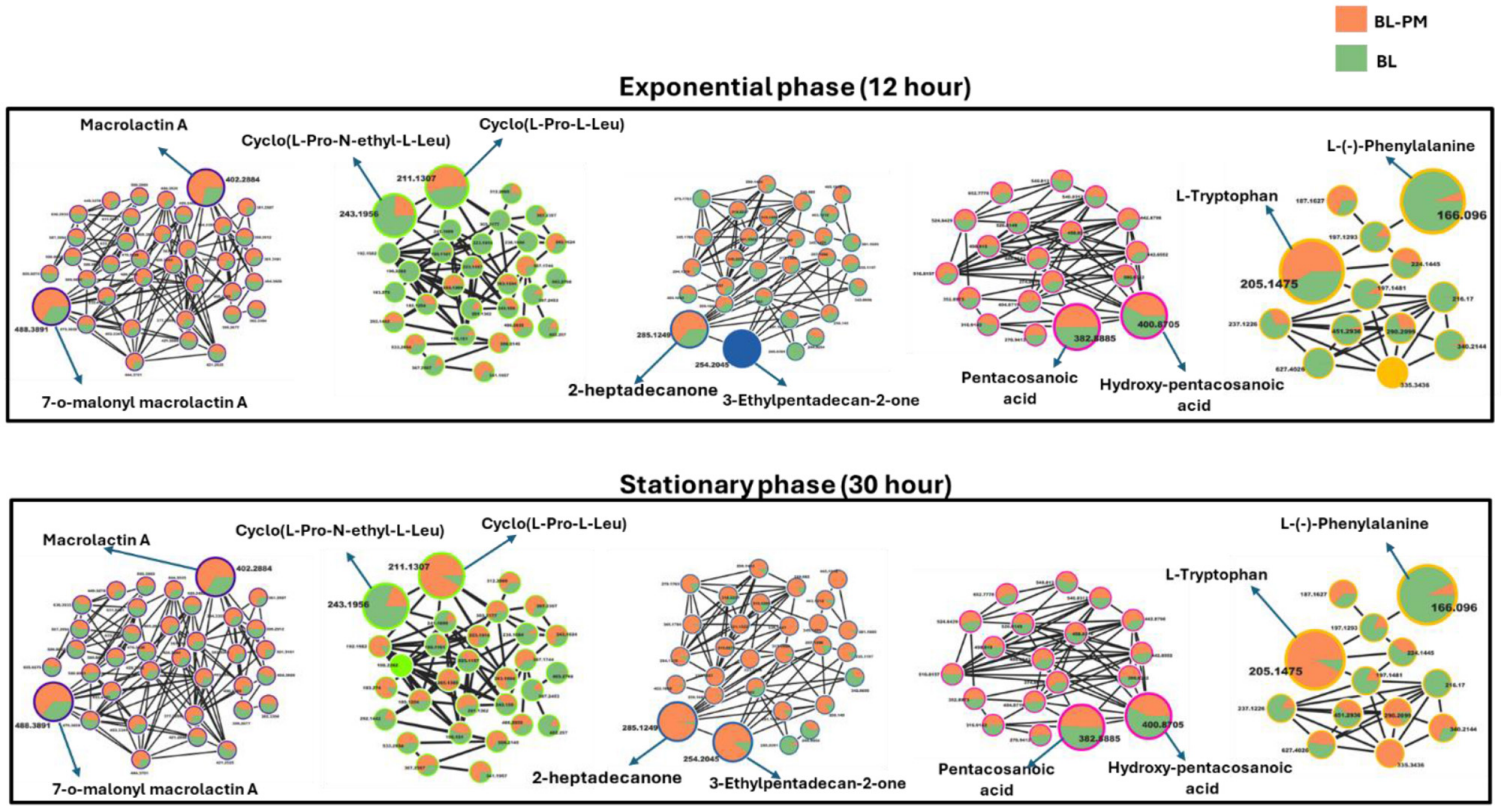
Molecular network of metabolites produced by *B. licheniformis* (BL) and co-culture with *P. megaterium* (BL-PM) at exponential and stationary phases. The molecular networks depict metabolite clusters at 12 h (Exponential phase) and 30 h (Stationary phase) for BL (orange nodes) and BL-PM (green nodes). Node size indicates whether a metabolite was annotated (larger nodes) or unannotated (smaller nodes), while the edges represent spectral similarity. The networks demonstrate the dynamic shifts in metabolite profiles between monoculture (BL) and treated (BL-PM) conditions. At the exponential phase, BL-PM shows enhanced production of macrolactins and amino acid derivatives, while BL predominantly produces saturated fatty acids. By the stationary phase, certain metabolites, such as hydroxy-pentacosanoic acid and cyclo(L-Pro-N-ethyl-L-Leu), show increased accumulation in co-culture conditions. This highlights the impact of microbial interaction and growth phase on metabolic production.

With the use of FBMN aided by Cystoscape, the superclasses of primary and secondary metabolites, including ketones and their derivatives, macrolides, cyclic peptides, saturated fatty acids, amino acids, and their derivatives, were annotated. Additionally, a thorough characterization of the measured metabolome was made possible by the molecular networking computation, which highlighted differences in the distribution of ions from different classes, as illustrated by the pie charts (Figures 4, 5), demonstrating the perturbations induced in one microbe by the culture extracts from another microbe. We examined the metabolic changes that occurred in *P. megaterium* and *B. licheniformis* during both the exponential (12 h) and stationary growth (30 h) phases, comparing treated and control samples. During the exponential phase (12 h), bacteria prioritize rapid cell division, leading to heightened metabolic activity geared toward growth and resource acquisition. This is evident in the control samples of *P. megaterium*, where cyclic peptides, ketones and amino acids were abundantly produced to support biosynthesis and energy demands (Figure 4).

However, in treated samples, these metabolites were reduced, possibly due to their depletion by *B. licheniformis*, or the presence of metabolites from *B. licheniformis* may have triggered an adaptive response in *P. megaterium*, leading to changes in primary and secondary metabolism. As *P. megaterium* transitioned into the stationary phase (30 h), the focus of its nutrient limitation shifted from growth to survival. This was marked by a decline in specific cyclic peptides, such as cyclo (L-Pro-L-Leu), as well as certain amino acids, like L-phenylalanine, and ketones, like 2-heptadecanone, in the control samples. The reduction of these metabolites suggests that *P. megaterium* may have downregulated biosynthetic pathways to conserve energy. In contrast, treated samples exhibited higher abundance in these compounds, indicating that exposure to *B. licheniformis* metabolites activates alternative metabolic pathways (Figure 4). Certain compound classes, including macrolides and fatty acids, displayed a relatively consistent distribution across both the exponential (12 h) and stationary growth (30 h) phases. However, within the macrolide group, specific compounds, such as macrolactin A, showed a higher accumulation in the control samples compared to the treated ones (Figure 4). This pattern suggests that while the overall presence of these compound classes remains stable throughout the bacterial growth cycle, individual compounds within each class can vary significantly, potentially influenced by external conditions in the growth environment or the metabolic and nutritional requirements of the bacteria at specific growth stages.

In *B. licheniformis*, cyclic peptides and amino acids were generally abundant during the exponential phase (12 h) in control samples compared to treated ones, suggesting their role in supporting rapid growth. However, in the stationary phase (30 h), metabolites such as cyclic peptides, ketones, and amino acids, including L-tryptophan, were significantly higher in the treated samples, indicating a shift in metabolic activity influenced by cross-feeding (Figure 5). This suggests that cross-feeding alters the timing and regulation of metabolite production. Additionally, macrolactins remained consistently higher in treated samples across both growth phases, possibly due to enhanced biosynthesis or prolonged stability (Figure 5). Together, these findings highlight the complex, treatment-dependent metabolic shifts in both *P. megaterium* and *B. licheniformis* across growth phases.

**Figure 6:**
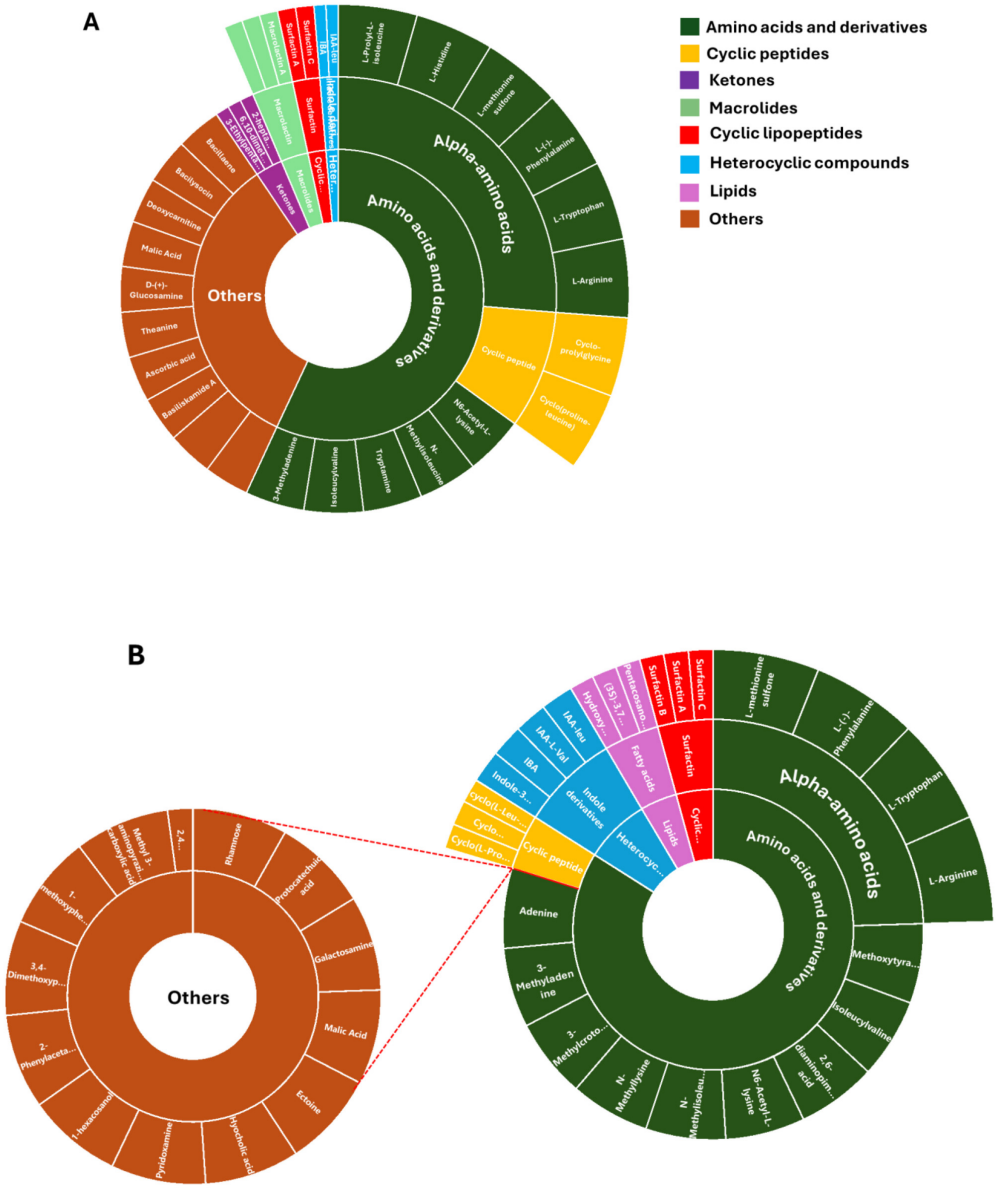
Metabolome coverage of *P. megaterium* **(A)** and *B. licheniformis* **(B)**. A sunburst plot illustrates the comprehensive set of metabolites identified through untargeted analysis from both treated and control samples. The visualization categorizes metabolites based on their chemical classes and abundance patterns. Detailed information, including the full names of the identified metabolites, is provided in Supplementary Table 3.

While FBMN remains highly beneficial, when used in conjunction with spectral library matching, it only facilitates the annotation of a small fraction of microbial MS/MS spectra (i.e., macrolactin A, cyclic peptides, ketones and derivatives, amino acids and fatty acids, Figure 3), highlighting a significant bottleneck in untargeted metabolomics analysis. The GNPS library databases contain mass spectra generated from different sample preparation techniques and mass spectrometers, resulting in variations in both the quality and content of the spectra. Furthermore, some of these spectra do not include chemical standards, which limits the ability to accurately annotate the metabolites. Consequently, manual verification is sometimes needed to confirm the tentative (semi-automated) annotations and predictions. Along with the FBMN metabolite annotations, metabolites including surfactin A, B, and C, Bacillaene, Bacilysocin, Basiliskamide A, Lichenysin-G6a, and 7-O-succinyl macrolactin F were manually annotated (Figure 6), further refining the metabolic profile of the studied microbial interactions.

### Time-dependent metabolic reprogramming reveals the key metabolite determinants influencing the interactions between *P. megaterium* and *B. licheniformis*

An interactive heatmap was used to quantitatively analyse the distribution of identified metabolites in control and treated samples at different time points. In addition to monitoring the bacteria's responses throughout time, this approach sought to visualize the effects of metabolites generated by one bacterium on the primary and secondary metabolism of another. Additionally, given that both species belonged to the *Bacillus* genus, distinguishing which metabolites originated from which species was challenging, which made it difficult to attribute specific metabolites to individual species. This is compounded by their genetic similarity and overlapping ecological niches (Grubbs et al., 2017). However, by utilizing a heatmap analysis, it became possible to determine whether specific metabolites are present and, if so, to assess their concentrations at different points. This method provides a clearer understanding of the metabolic profiles of each species, enabling the differentiation of their contributions to the overall metabolite pool.

**Figure 7:**
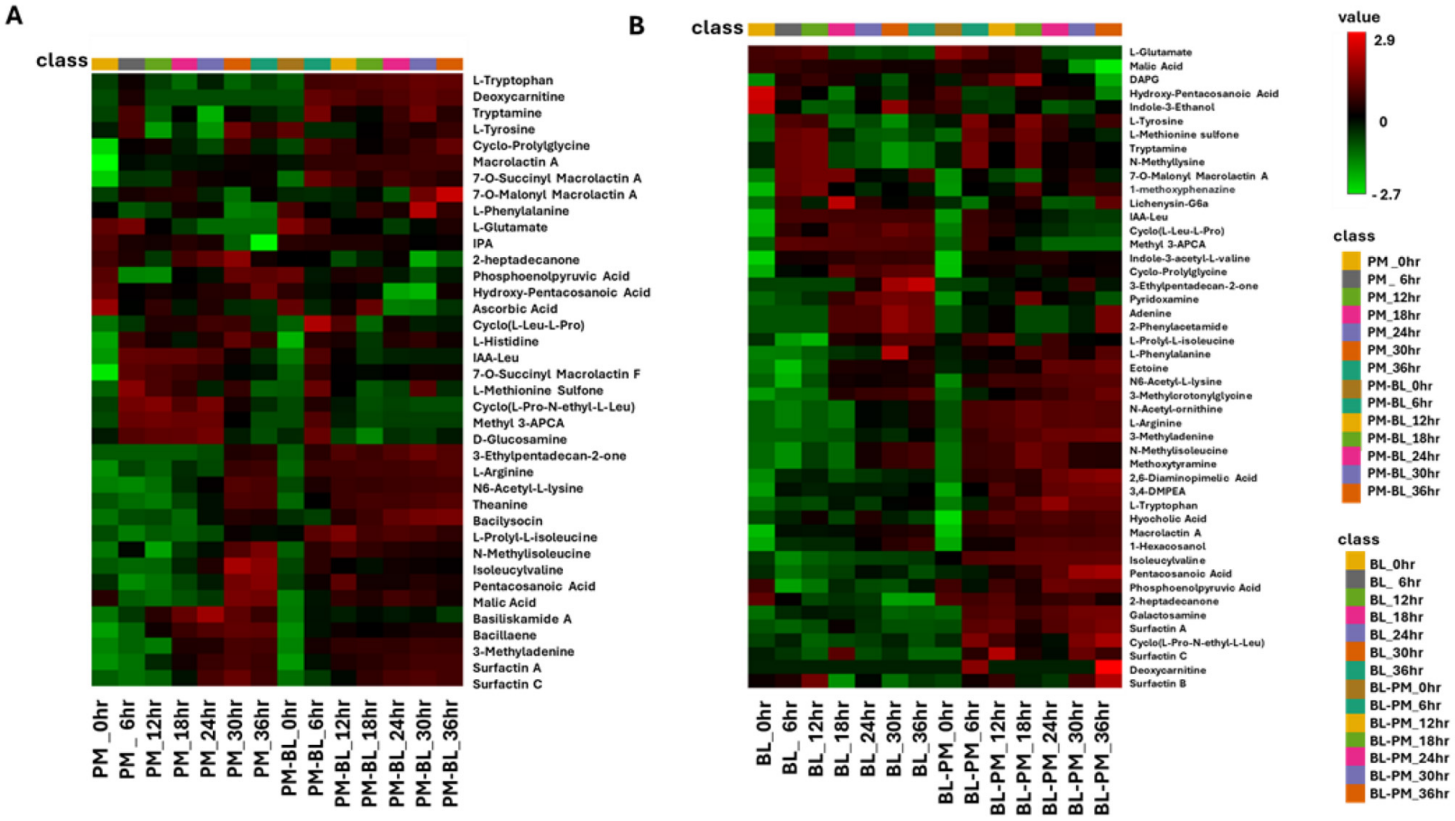
The interactive heatmap analysis depicts the profiles of tentatively annotated metabolites in control and treated samples of *P. megaterium* and *B. licheniformis* across a time course from 0 to 36 h. **(A)** Control (*P. megaterium*, PM) and treated samples (PM-BL), **(B)** control (*B. licheniformis*, BL) with treated samples (BL-PM). Samples and time points are represented in columns, while metabolites are arranged in rows. The data were log-transformed and *Pareto*-scaled using MetaboAnalyst for improved visualization. The heatmap uses a color gradient, where red indicates high abundance and green signifies low abundance, to highlight metabolite dynamics. Notably, some metabolites exhibit high abundance in specific treated samples but low abundance in others, indicating the perturbations induced by metabolites from other bacteria. Abbreviations: hydroxy-pentacosanoic acid (25-Hydroxy-C25:0), indole-3-acetyl-L-leucine (IAA-leu), methyl 3-aminopyrazine-2-carboxylic acid (Methyl 3-APCA), indole-3-acetyl-L-valine (IA-L-Val), 3-methylcrotonylglycine (3-MCG), 2,6-diaminopimelic acid (2.6 DAP), 3,4-dimethoxyphenethylamine (3,4-DMPEA), and 1-methyl-2-ubiquinone (1-Me-2-Ud-QnO).

The interactive heatmap analysis of *P. megaterium* samples (Figure 7) and *B. licheniformis* samples revealed distinct patterns of metabolite regulation over the 36-h period, comparing treated samples with control samples. In *P. megaterium*, cross-feeding resulted in an increase (red color) in primary metabolites, such as L-tryptophan, deoxycarnitine, tryptamide, and isoleucyvaline,among others. These changes reflect a shift in amino acid metabolism, energy production, and the potential biosynthesis of secondary metabolites, indicating that cross-feeding supports growth and interspecies communication. Additionally, secondary metabolites such as 3-ethylpentadecan-2-one, bacilysocin, pentacosanoic acid, surfactin A and B also showed increased (red color), suggesting a significant metabolic reprogramming triggered by exposure to other bacterial metabolites.

In *B. licheniformis*, cross-feeding with metabolites from *P. megaterium* led to a significant shift in metabolite production, marked by a notable increase (red color) in several primary metabolites (such as L-tryptophan, L-arginine, and 3-methyladenine) and secondary metabolites like cyclic lipopeptides (including surfactin A and C), and fatty acids (such as pentacosanoic acid and phosphoenolpyruvic acid). These changes suggest that *B. licheniformis* is adapting to the presence of external metabolites, potentially to enhance growth, improve stress tolerance, or meet specific functional needs in the altered metabolic environment. The increase in amino acids and fatty acids could be linked to cellular processes such as protein synthesis, membrane integrity, and energy production, while the elevated cyclic lipopeptides may indicate a response to microbial competition or an enhancement of motility and biofilm formation. In contrast, there was a decrease (green color) in the levels of cyclic peptides, compounds generally associated with antimicrobial activity or cell signaling. However, certain metabolites, such as N-methylisoleucine and methoxytyramine, remained consistent at both 30 and 36 h in both the control and treated samples. This consistency suggests that these metabolites may serve as core components of the bacterium's metabolism, essential for maintaining fundamental cellular functions regardless of the environmental perturbations induced by cross-feeding.

**Figure 8:**
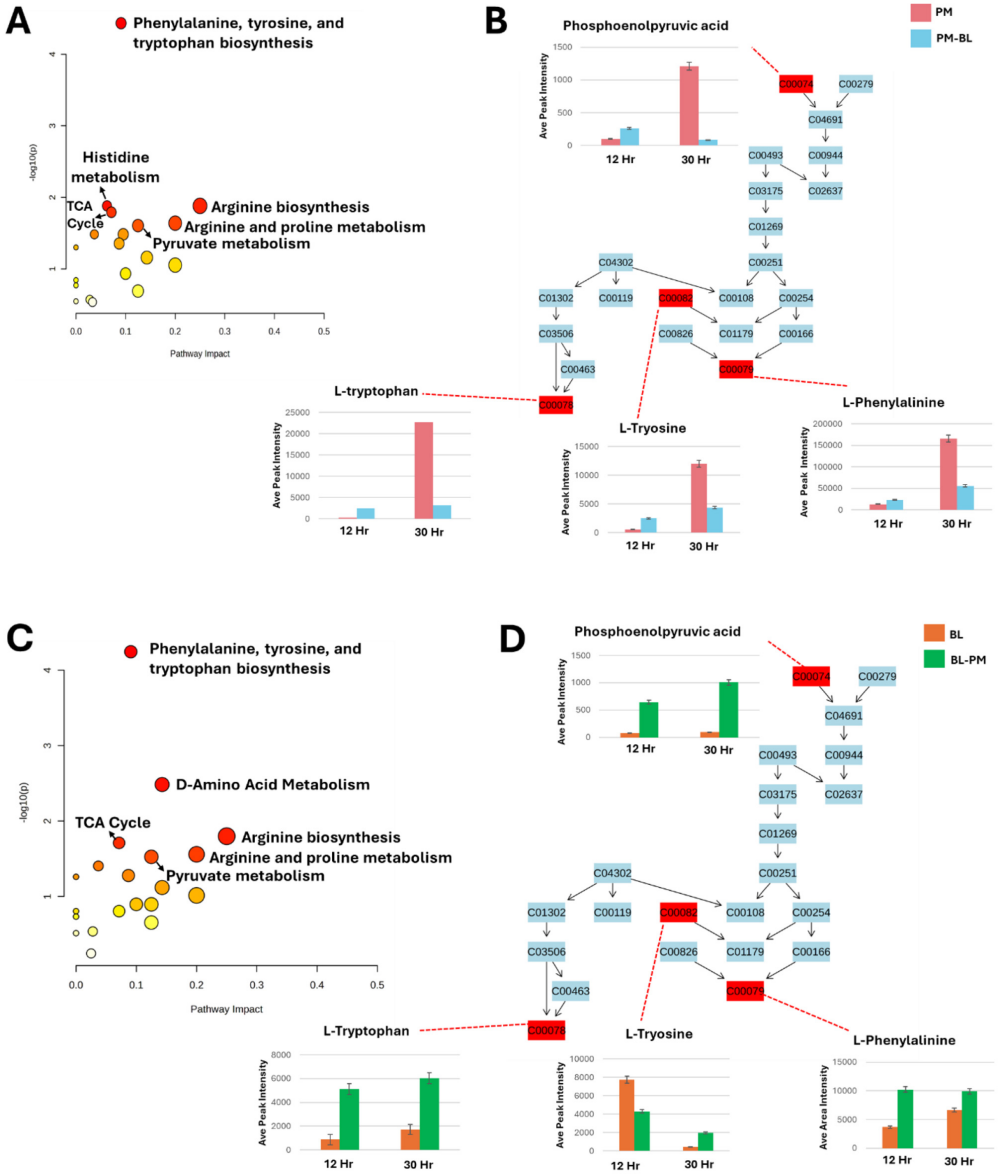
Metabolic pathway analysis and relative quantification of altered metabolites in the phenylalanine, tyrosine, and tryptophan biosynthesis pathway. **(A, C)**: Topological representation for *P. megaterium* (PM) **(A)** and *B. licheniformis* (BL) **(C)**, highlighting statistically significant pathways identified from the metabolome view based on matched metabolites. The pathways are ranked according to the *p*-value (y-axis) from enrichment analysis and pathway impact values (x-axis) derived from pathway topology analysis. The node color reflects the p-value (red = lowest *p*-value and highest statistical significance), while node size corresponds to the pathway impact factor, with larger nodes indicating higher impact. **(B, D)**: Pathway analysis of the phenylalanine, tyrosine, and tryptophan biosynthesis (PTTB) pathway for PM **(B)** and BL **(D)**. These panels display the relative quantification levels (averaged peak areas) of mapped metabolites during the exponential and stationary phases.

The metabolites listed in Supplementary Table 3 were analyzed using MetaboAnalyst Pathway Analysis (MetPA) to identify key metabolic pathways associated with the data for both species under treated and control conditions (Figure 8). The pathways are arranged based on the *p-*value (y-axis), which indicates the pathway enrichment analysis and pathway impact values (x-axis) representing pathway topology analysis. In *P. megaterium*, the most significant pathways indicated by a high *p*-value on the y-axis were the phenylalanine, tyrosine, and tryptophan biosynthesis (PTTB) pathway, followed by the histidine biosynthesis pathway (Figure 8A). This latter pathway plays a crucial role in bacterial interactions by supporting growth, survival, and communication. Histidine is an essential amino acid that serves as a precursor for protein synthesis and energy production, enabling bacteria to thrive in nutrient-limited environments and adapt to competitive microbial ecosystems. However, the observed metabolite profiles in the heatmaps suggest a negative correlation between the impacted metabolic pathways and histidine production, indicating that cross-feeding with *B. licheniformis* metabolites may have disrupted histidine biosynthesis in *P. megaterium*. This metabolic suppression could have directly contributed to the reduced growth of *P. megaterium* under these conditions, as histidine is essential for cellular functions, including enzyme activity, stress response, and metabolic regulation.

Similarly, in *B. licheniformis* the PTTB pathway was the most prominent followed by D-amino acid metabolism pathway (Figure 8B), which is essential for bacterial physiology, facilitating the conversion of L-amino acids into their D-enantiomers, which are critical for various cellular processes and also play a key role in peptidoglycan synthesis, where they contribute to cell wall remodeling and structural integrity, thereby enhancing bacterial resilience during interactions with other microbes. In the observed heatmap data, there was an increase (green color) in L-amino acids, indicating a positive correlation between the levels of L-amino acids and the D-amino acid pathway. This suggests that the increased availability of L-amino acids (green color) may have fuelled the enzymatic conversion to D-forms. This relationship highlights the dynamic interplay between amino acid metabolism and bacterial adaptation, potentially influencing cell wall composition, signaling mechanisms in cross-feeding environments.

In this section, we focused on the (PTTB) pathway, as it appeared to have the lowest *p*-value (< 0.1) for both *P. megaterium* and *B. licheniformis* (Figures 8A,C). This pathway is fundamental to bacterial metabolism, serving as a cornerstone for growth, survival, and adaptation. Aromatic amino acids derived from this pathway are not only essential for protein synthesis, contributing to the structure and function of enzymes and structural proteins, but also play a crucial role in the biosynthesis of secondary metabolites. These metabolites are crucial to bacterial adaptation, offering advantages such as stress resistance and enhanced interaction within complex microbial communities. To gain deeper insights into metabolic perturbations, we examined changes occurring at two critical growth phases: the exponential phase (12 h), when bacteria exhibit high metabolic activity and resource acquisition, and the stationary phase (30 h), where metabolic shifts favor survival strategies in response to nutrient depletion (Figures 8B,D).

**Figure 9:**
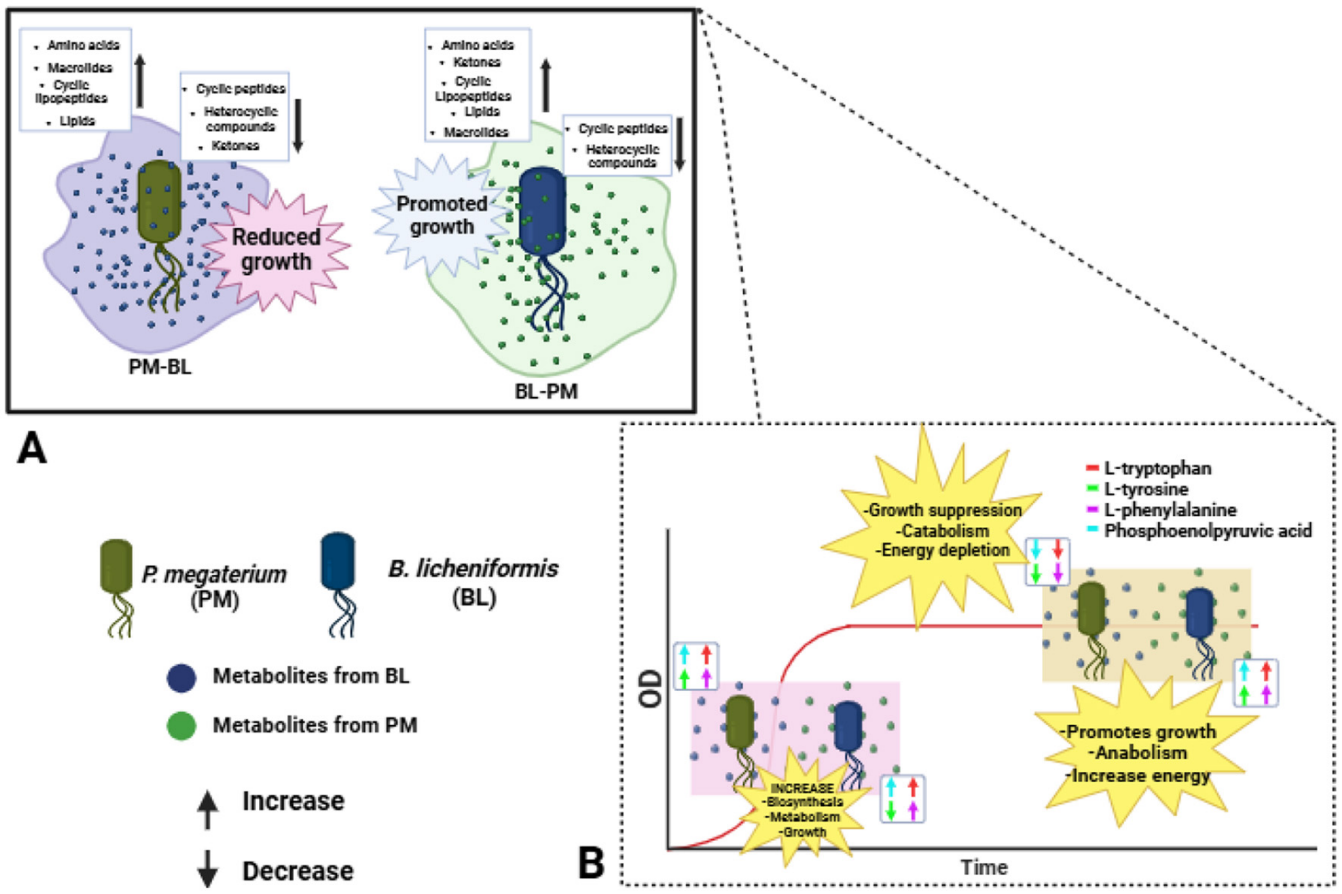
This summary examines the metabolic and growth responses of *P. megaterium* (PM) and *B. licheniformis* (BL) during cross-feeding interactions. **(A)** Overview of perturbations induced by cross-feeding; PM showed increased production of amino acids, macrolides, cyclic lipopeptides, and lipids, but experienced growth suppression due to decreased levels of cyclic peptides, heterocyclic compounds, and ketones. Conversely, BL grown with PM metabolites exhibited enhanced growth, characterized by increased amino acids, ketones, cyclic lipopeptides, lipids, and macrolides. **(B)** The underlying effect of growth is induced by primary metabolites. During the exponential phase (12 h), both species benefited from elevated levels of key metabolites, including L-tryptophan, L-tyrosine, and L-phenylalanine. However, during the stationary phase, PM experienced growth suppression and energy depletion, whereas BL maintained its growth and energy balance.

To summarize the metabolic findings in detail, we utilized an illustrative figure to effectively convey the results of our study (Figure 9). *P. megaterium* showed an increase in the production of amino acids, macrolides, cyclic lipopeptides and lipids. However, this increase was accompanied by a decrease in cyclic peptides, heterocyclic compounds, and ketones. The same was observed in *B. licheniformis*; however, it showed reduced levels of cyclic peptides and heterocyclic compounds. To gain a deeper understanding of these changes, we examined the biosynthesis pathway for phenylalanine, tyrosine, and tryptophan. During the exponential phase (12 h), both species exhibited increased levels of key metabolites, including L-tryptophan, L-tyrosine, L-phenylalanine, and phosphoenolpyruvic acid, indicating enhanced growth and biosynthesis. However, during the stationary phase (30 h), PM experienced a decline in these metabolites, which may lead to metabolic dysregulation and growth suppression. In contrast, BL displayed an increase in these metabolites during the stationary phase (30 h), suggesting improved growth and energy availability. Overall, the metabolic dynamics differ significantly between the two species across growth phases.

## Discussion

PGPR are frequently referred to as “the second plant genome” due to their importance in plant growth, development, nutrition, secondary metabolite biosynthesis, and defense. However, for PGPR to carry out their function at maximum capacity, they typically interact with one another within their environment by secreting a variety of compounds, including signal molecules, antibiotics, and metabolic byproducts (Wang et al., 2012). These compounds play a significant role in shaping the growth, activity, and survival of adjacent microbes, creating a dynamic network in the rhizosphere that promotes both microbial diversity and overall plant growth, development and health (Wannaporn and Dusit, 2024).

### Cross-feeding between *P. megaterium* and *B. licheniformis* influences their growth and interaction

To examine how metabolites produced by one PGPR affect neighboring PGPR, we used minimal media for cultivation. This approach was chosen because minimal media creates a controlled environment with restricted nutrients, enabling us to more effectively observe the effects of metabolites generated during bacterial growth. By reducing external influences, the interaction between the metabolites and the bacteria became clearer. Analysis of growth curves revealed that culture extracts from *B. licheniformis* significantly reduced the growth of *P. megaterium* (Figure 1A). This might be because the metabolites produced by *B. licheniformis* during its stationary phase (30 h) have inhibitory or non-essential nutritional effects on *P. megaterium*.

Additionally, the competitive dynamics between these two species may play a crucial role in this observed inhibition. As *B. licheniformis* proliferates and produces metabolites, it may create an environment that favors its own growth while limiting that of *P. megaterium*. This interaction could be a result of co-evolutionary strategies where *B. licheniformis* has developed mechanisms to outcompete neighboring microbes for resources, as highlighted by Pacheco et al. (2019). In this context, *B. licheniformis* may deploy these metabolites not only for its benefit but also to establish dominance within the microbial community. This highlights an important consideration in microbial formulations as bio-stimulants. While combining two or more PGPR strains for plant inoculation can enhance plant growth, the interactions between these strains can lead to variable outcomes. The observed changes in metabolite production suggest that microbial co-culturing does not always result in additive or synergistic effects; rather, it can lead to a shift in metabolic priorities that may enhance, neutralize, or even inhibit the growth and function of one strain over the other.

The growth-promoting effect of *P. megaterium* metabolites on *B. licheniformis* can be attributed to the differences between the rates of metabolite formation and conversion (Figure 1B). As Pinu et al. (2017) explained, the concentration of each metabolite present in a microbial cell is determined by this balance; when the rate of production exceeds that of conversion, metabolites accumulate and positively influence the growth of neighboring species. In this case, *P. megaterium* likely produces metabolites that may serve as precursors for vital biosynthetic pathways or provide energy sources that enhance various physiological processes in *B. licheniformis*, such as nutrient uptake and cellular metabolism.

### Cross-feeding induces metabolic shifts in *P. megaterium* and *B. licheniformis* across growth phases

During the exponential growth phase, bacterial species are known to exhibit high metabolic activity to support rapid cell division, usually utilizing glycolysis and pentose phosphate pathway (PPP) to process glucose (Godard et al., 2020), which are central to bacterial energy production and biosynthesis during exponential growth phases (Biedendieck et al., 2021). This phase is characterized by the production of a variety of metabolites, including amino acids, fatty acids and hydrocarbons, which aid in biosynthesis and stress response. As glucose is processed, excess metabolites, such as acetate and lactate, accumulate, resembling the Crabtree effect observed in E. coli (Alberto et al., 2012). Once glucose is exhausted, these organic acids serve as secondary carbon sources. As bacterium transitions to the stationary phase due to nutrient scarcity, it downregulates non-essential pathways to conserve resources. This metabolic shift redirects energy toward cellular maintenance rather than growth, a common survival tactic for bacteria.

During the exponential phase (12 h), the FBMN revealed molecular changes occurring during cross-feeding, particularly a noticeable reduction in the levels of cyclic peptides and amino acids in treated samples of both *P. megaterium* and *B. licheniformis* (Figures 4, 5). This decline suggests that these microbes may have depleted these metabolites from their media either through direct consumption or by producing compounds that modified the metabolic environment, thereby limiting their availability. This phenomenon is similar to what occurs in clonal populations of *B. subtilis*, where different subpopulations specialize in producing compounds like acetate or acetoin (Rosenthal et al., 2018). The accumulation of acetate by one subpopulation influences the environment, which in turn affects the metabolic behavior and growth of other subpopulations (Rosenthal et al., 2018). As the bacteria transitioned into the stationary phase (30 h), both *P. megaterium* and *B. licheniformis* exhibited an increase in ketones (Figures 4, 5), suggesting a shift in metabolic activity toward survival and adaptation under nutrient-limiting conditions. Ketones play a role in alternative energy metabolism, acting as intermediates in lipid catabolism and stress response pathways. Their increased accumulation may indicate an adaptive strategy to maintain cellular function in response to metabolic constraints imposed by cross-feeding interactions. Additionally, *B. licheniformis* displayed a notable increase in certain fatty acids during this phase (Figure 5). Fatty acids are essential for maintaining membrane integrity, modulating membrane fluidity, and contributing to the biosynthesis of secondary metabolites. Their elevated production in *B. licheniformis* could be a response to environmental stress, suggesting that *B. licheniformis* may utilize fatty acid metabolism as one of its survival mechanisms, thereby reinforcing its cellular defense in an altered metabolic environment.

### Time-dependent metabolic reprogramming and its role in cross-feeding interactions between *P. megaterium* and *B. licheniformis*

Time-dependent metabolic reprogramming revealed key metabolite determinants responsible for perturbations induced by cross-feeding interactions between *P. megaterium* and *B. licheniformis*. In both treated samples, a notable increase in amino acids, macrolides, and cyclic lipopeptides was observed (Figure 7). These compounds are known for their ability to modulate microbial interactions, survival, and exhibit antimicrobial properties, which are crucial in competitive microbial environments (Dai et al., 2022; Zhang et al., 2023). Interestingly, this metabolic shift highlights the intricate relationship between primary and secondary metabolites during cross-feeding. The primary metabolites, such as amino acids (L-tyrosine, L-tryptophan, and L-arginine), among others, play a fundamental role in supporting the growth and maintenance of cellular functions in microorganisms by serving as building blocks for proteins and driving essential metabolic processes (Liu et al., 2020; Dai et al., 2022).

However, beyond their primary roles, these metabolites also modulate the production of secondary metabolites, such as macrolactin A, 7-O-succinyl macrolactin A, surfactin A, B, and C, bacilysocin, 2-heptadecanone, and pentacosanoic acid. Secondary metabolites, unlike primary metabolites, are not directly involved in growth but serve critical ecological functions that enhance microbial survival and competitiveness. For instance, the increased levels of macrolides and cyclic lipopeptides (Figure 7) observed in samples may provide a competitive advantage by inhibiting the growth of nearby microorganisms, including harmful ones, disrupting biofilms and improving nutrient acquisition. In addition to the other findings, time-dependent metabolic reprogramming highlighted an increase in antimicrobial compounds in both species from treated samples compared to control samples. This was accompanied by a decrease in non-toxic compounds such as cyclic peptides and heterocyclic compounds. This shift is likely because many bacteria have developed mechanisms to convert relatively non-toxic compounds into highly toxic ones (Chen and Rosen, 2016; Chen et al., 2019; Yan et al., 2019), thereby creating antimicrobial stress for susceptible bacteria. This adaptive capability allows bacteria to enhance their competitive advantage in environments where antimicrobial pressure is present, potentially leading to a more complex microbial ecosystem where only the most resilient bacteria can thrive. The antimicrobial compounds had a minimal impact on the growth of *B. licheniformis* compared to *P. megaterium*, largely due to the ability of some *Bacillus* species to tolerate or detoxify antimicrobial substances. For example, certain strains like *Bacillus sp. BP-3* can detoxify antimicrobial compounds by using enzymes such as *ArsM*, which methylates these compounds into less toxic forms (Ge et al., 2023). This detoxification capability not only ensures the survival of cross-feeding strains but also provides them with a competitive advantage by allowing them to exploit resources like arsenic in microbial competition (Ge et al., 2023).

### The influence of primary and secondary metabolites via PTTB pathway on microbial cross-feeding

Microbial interactions are significantly influenced by the production and availability of secondary metabolites, which are often dependent on primary metabolites. For instance, primary metabolites from PTTB (Figure 8B) serve as precursors for compounds like flavonoids, alkaloids, indoles, among others, and they play a significant role in signaling and survival among bacterial species (Lozano et al., 2019; Bao et al., 2022; Gokul Anil Kumar et al., 2024). During the exponential phase (12 h), the increased production of primary metabolites in the treated *P. megaterium* (Figure 8B) enables effective bacterial signaling and communication, supporting cooperative behaviors such as biofilm formation and resource sharing. These metabolites also drive the biosynthesis of secondary metabolites, which serve as stress protectants, enhancing the bacterium's adaptability to environmental changes (Zhang et al., 2024). However, in the stationary phase (30 h), their suppression limits protein and secondary metabolite synthesis, which may also reflect an adaptive stress rather than a simple inhibition of metabolism. Such reprogramming could extend beyond bacterial survival, as the reduced abundance of aromatic amino acid-derived metabolites also may interfere with *P. megaterium*'s auxin production capacity, potentially reducing its ability to interact effectively with plant roots and access plant-derived exudates (Moormann et al., 2022). This metabolic decline may induce a persistent state, which is a survival strategy that enables endurance under unfavorable conditions but restricts active growth (Amato et al., 2014), ultimately affecting *P. megaterium*'s resilience and ecological success.

In contrast, the treated *B. licheniformis* exhibited sustained production of primary metabolites across both the exponential (12 h) and stationary phases (30 h) (Figure 8D). This suggests a prolonged metabolic activity that may provide continuous support for cellular functions, stress adaptation, and microbial interactions even as growth slows. The persistence of primary metabolites in *B. licheniformis* could enhance its ecological competitiveness by maintaining metabolic exchange and cooperative behaviors over an extended period. This also explains why growth suppression was observed in *P. megaterium*, while *B. licheniformis* exhibited growth enhancement. Since *P. megaterium* relies on a transient increase in primary metabolites during the exponential phase (12 h), the rapid depletion of these metabolites in the stationary phase (30 h) likely limits essential cellular functions, secondary metabolite production, and metabolic exchange.

Moreover, despite belonging to the same genus, *B. licheniformis* and *P. megaterium* exhibit other distinct metabolic pathways that reflect their adaptations to different ecological niches. *P. megaterium* is also known to utilize the Embden-Meyerhof-Parnas (EMP) pathway for glycolysis and the oxidative pentose phosphate (OPP) pathway for carbohydrate catabolism, lacking the Entner-Doudoroff (ED) pathway (Wushensky et al., 2018). In contrast, *B. licheniformis* can employ alternative pathways, including the ED pathway under certain conditions, allowing it to thrive on a broader range of carbon sources compared to *P. megaterium*. This adaptability, combined with detoxification mechanisms, enhances their survival and competitiveness in various environments.

While both *P. megaterium* and *B. licheniformis* can coexist within similar habitats, their differing metabolic strategies significantly influence their ecological roles, competitive abilities, and contributions to microbial community dynamics. The contrasting metabolic approaches of these two species highlight their unique functions within these communities. *P. megaterium* may contribute to specific ecological functions through its secondary metabolites during favorable conditions, enhancing interactions and signaling within the microbial ecosystem. On the other hand, *B. licheniformis* appears better equipped to thrive in diverse environments due to its sustained production of primary metabolites, which not only support its growth but also enhance its ability to acquire nutrients and adapt to varying ecological pressures. Together, these dynamics illustrate the intricate balance of competition and cooperation that shapes microbial community structures and functions. Importantly, the asymmetric growth response, with *B. licheniformis* benefiting from *P. megaterium* metabolites but not vice versa, underscores the relevance of interspecies metabolic compatibility in co-formulating PGPR inoculants. This challenges the assumption that co-inoculation always yields additive or synergistic benefits and emphasizes the need for metabolic compatibility testing during bioinoculant development.

Furthermore, the induction of secondary metabolites, such as surfactins, macrolactins, and bacilysocin, during cross-feeding suggests a microbial strategy for competitive exclusion or niche adaptation. These findings are not only valuable for microbiome engineering but also have broader implications for the design of microbial consortia in industrial fermentation, soil remediation, and climate-resilient agriculture. From a sustainability perspective, this work directly supports sustainable development in the form of SDG 12 (responsible consumption and production) by advancing the development of metabolically compatible, efficient microbial formulations that reduce dependency on chemical inputs; SDG 13 (climate action) by promoting biological alternatives that enhance soil carbon cycling and stress resilience; SDG 9 (industry, innovation, and infrastructure) by informing biotechnological innovation in microbial bioformulation; and indirectly, SDG 3 (good health and wellbeing) by reducing the environmental footprint of agrochemical overuse and dependency.

## Conclusion

This study highlights the complex metabolic interactions between two PGPR, revealing how the production of primary and secondary metabolites influences microbial cross-feeding dynamics. The results demonstrate that while *P. megaterium* exhibits a transient increase in primary metabolite production during the exponential phase (12 h); its metabolic activity declines in the stationary phase (30 h), leading to growth suppression. In contrast, *B. licheniformis* maintains sustained production of primary metabolites, thereby enhancing its adaptability and competitive advantage in various environments. Moreover, the observed metabolic shifts suggest that cross-feeding interactions can significantly alter bacterial physiology, promoting either cooperative or competitive outcomes, depending on the environmental conditions. The differential metabolic strategies of these two species not only shape their individual survival but also contribute to the broader structure of the microbial community. These findings underscore the importance of metabolic plasticity in microbial ecology and may have implications for optimizing microbial formulations in agricultural and biotechnological applications. Future studies integrating transcriptomic sequencing with metabolomic profiling will provide a comprehensive understanding of the gene expression changes underlying the observed metabolic reprogramming in bacterial cross-feeding interactions. These studies could further explore the molecular mechanisms regulating these interactions and their potential applications in sustainable agriculture and bioengineering. Understanding the precise metabolic cues that mediate microbial cross-feeding could facilitate the development of tailored microbial consortia for enhanced plant growth and soil health in supporting sustainable production systems (SDG 12), enhancing agroecosystem resilience (SDG 13), and contributing to innovation in microbial biotechnology (SDG 9).

## Data Availability

The original contributions presented in the study are included in the article/supplementary material, further inquiries can be directed to the corresponding authors.
