# Supplementary material for: Metabolic reprogramming in PGPR reveals cross-feeding-driven physiological shifts and metabolic adaptations
Source: Front Microbiol. 2025 Nov 24;16:1668025. doi: 10.3389/fmicb.2025.1668025 (PMC12683585; doi:10.3389/fmicb.2025.1668025)
Supplement: Supplementary file 1 [file Supplementary_file_1.docx]

**Kamogelo Mmotla^1,2^, Farhahna Allie^1^, Thendo Mafuna^1^, Manamela D Mashabela^1,2,3*^ and Msizi I Mhlongo^1,2,3*^**

^1^Department of Biochemistry, Faculty of Science, University of Johannesburg, Auckland Park, 2006, South Africa.

^2^Imbewu Metabolomics Research Group, Department of Biochemistry, Faculty of Science, University of Johannesburg, Auckland Park Kingsway Campus, P.O. Box 524, Johannesburg 2006, South Africa.

^3^Research Centre for Plant Metabolomics, Faculty of Science, University of Johannesburg, Auckland Park, 2006, South Africa
*Correspondence: E-mail: [manamelem@uj.ac.za](mailto:manamelem@uj.ac.za); [mmhlongo@uj.ac.za](mailto:mmhlongo@uj.ac.za); Tel.: +27-11-559-4573

# **Supplementary files**

**Table S1: The optical density (OD_600_) measurements for PM:** Monocultured PM, served as the control, and PM cultured in metabolites produced by BL used as the experimental group. These measurements were taken at various time intervals (0-36 hr), allowing for a detailed comparison of PM growth dynamics under each condition.

| **Microbes** | **Time** | **Rep 1** | **Rep 2** | **Rep 3** | **Average** |
| --- | --- | --- | --- | --- | --- |
| **PM** | 0 | 0.01 | 0.01 | 0.01 | 0.01 |
| **PM** | 6 | 0.66 | 0.055 | 0.023 | 0.246 |
| **PM** | 12 | 1.307 | 1.383 | 1.243 | 1.311 |
| **PM** | 18 | 1.692 | 1.821 | 1.817 | 1.776667 |
| **PM** | 24 | 1.836 | 1.775 | 1.85 | 1.820333 |
| **PM** | 30 | 1.649 | 1.987 | 1.868 | 1.834667 |
| **PM** | 36 | 1.768 | 1.935 | 1.714 | 1.805667 |
|  |  |  |  |  |  |
| **PM-BL** | 0 | 0.01 | 0.01 | 0.01 | 0.01 |
| **PM-BL** | 6 | 0.062 | 0.061 | 0.075 | 0.066 |
| **PM-BL** | 12 | 0.516 | 0.685 | 0.512 | 0.571 |
| **PM-BL** | 18 | 0.622 | 0.769 | 0.708 | 0.699667 |
| **PM-BL** | 24 | 0.809 | 0.606 | 0.556 | 0.657 |
| **PM-BL** | 30 | 0.775 | 0.511 | 0.667 | 0.651 |
| **PM-BL** | 36 | 1.766 | 1.42 | 0.52 | 1.235333 |

**Table S2: The optical density (OD_600_) measurements for BL:** Monocultured BL, served as the control, and BL cultured in metabolites produced by PM (ME) used as the experimental group. These measurements were taken at various time intervals (0-36 hr), allowing for a detailed comparison of BL growth dynamics under each condition.

| **Microbes** | **Time** | **Rep 1** | **Rep 2** | **Rep 3** | **Average** |
| --- | --- | --- | --- | --- | --- |
| **BL** | 0 | 0.1 | 0.1 | 0.1 | 0.1 |
| **BL** | 6 | 0.196 | 0.195 | 0.2 | 0.197 |
| **BL** | 12 | 0.473 | 0.548 | 0.602 | 0.541 |
| **BL** | 18 | 0.701 | 0.495 | 0.637 | 0.611 |
| **BL** | 24 | 0.628 | 0.781 | 0.731 | 0.713333 |
| **BL** | 30 | 0.722 | 0.721 | 0.781 | 0.741333 |
| **BL** | 36 | 0.902 | 0.835 | 0.894 | 0.877 |
|  |  |  |  |  |  |
| **BL-PM** | 0 | 0.1 | 0.1 | 0.1 | 0.1 |
| **BL-PM** | 6 | 0.363 | 0.309 | 0.329 | 0.333667 |
| **BL-PM** | 12 | 0.39 | 0.352 | 0.695 | 0.479 |
| **BL-PM** | 18 | 0.695 | 0.214 | 0.396 | 0.435 |
| **BL-PM** | 24 | 0.336 | 0.888 | 0.344 | 0.522667 |
| **BL-PM** | 30 | 0.328 | 0.718 | 0.395 | 0.480333 |
| **BL-PM** | 36 | 0.412 | 0.388 | 0.369 | 0.389667 |

**Table S3:** **An overview of the annotated and tentatively identified metabolites from treated and controlled samples of PM and BL.** The tick mark (**✔**) indicates the metabolite was detected in the specific sample, while a dash (**X**) signifies it was not identified. The classification of these metabolites is visually displayed in the sunburst plot (**Figure 3.6**), while their qualitative and quantitative distribution is illustrated in the heatmap (**Figure 3.7**).

| **No** | **Compound name** | **Formula** | **rt (min)** | **m/z** | **Fragments** | **Adduct** | **BL** | **BL-PM** | **PM** | **PM-BL** |
| --- | --- | --- | --- | --- | --- | --- | --- | --- | --- | --- |
| 1 | 2,4-diacetylphloroglucinol | C_10_H_10_O_5_ |  | 210.0756 | 193 | M+H^+^ | ✔ | ✔ | X | X |
| 2 | Theanine | C_7_H_14_N_2_O_3_ | 0.86 | 197.0439 | 179,508 | [M+Na]^+^ | X | X | ✔ | ✔ |
| 3 | Galactosamine | C_6_H_13_NO_5_ | 0.92 | 180.0969 | 142, 162 | [M+H]^+^ | X | ✔ | ✔ | ✔ |
| 4 | Hydroxy-pentacosanoic acid | C_25_H_48_O_3_ | 0.96 | 400.8705 |  | [M+H]^+^ | ✔ | ✔ | ✔ | X |
| 5 | L-Arginine | C_6_H_14_N_4_O_2_ | 1.01 | 175.1221 | 116, 130, 158 | [M+H]^+^ | ✔ | ✔ | ✔ | ✔ |
| 6 | N-Acetyl-ornithine | C_7_H_14_N2O_3_ | 1.011 | 175.1221 | 43,115 | [M+H]^+^ | ✔ | ✔ | X | X |
| 7 | Malic Acid | C_4_H_6_O_5_ | 1.06 | 157.0245 | 149, 142 | [M+Na]^+^ | ✔ | ✔ | ✔ | ✔ |
| 8 | Pentacosanoic acid | C_25_H_50_O_2_ | 1.10 | 382.8885 | 100,382 | [M+H]^+^ | ✔ | ✔ | ✔ | ✔ |
| 9 | Adenine | C_5_H_5_N_5_ | 1.31 | 136.0706 | 100,137 | [M+H]^+^ | ✔ | ✔ | X | X |
| 10 | 2-Phenylacetamide | C_8_H_9_NO | 1.32 | 136.0706 | 118 | [M+H]^+^ | ✔ | ✔ | X | X |
| 11 | 2,6-diaminopimelic acid | C_7_H_14_N_2_O_4_ | 1.57 | 191.103 | 140 | [M+H]^+^ | X | ✔ | X | X |
| 12 | L-methionine sulfone | C_5_H_11_NO_4_S | 1.62 | 182.0536 | 99 | [M+H]^+^ | ✔ | ✔ | ✔ | ✔ |
| 13 | 3-Methylcrotonylglycine | C_7_H_11_NO_3_ | 1.82 | 158.0892 | 99, 141 | [M+H]^+^ | ✔ | ✔ | X | X |
| 14 | 3-Methyladenine | C_6_H_7_N_5_ | 1.99 | 150.0839 | 151 | [M+H]^+^ | ✔ | ✔ | ✔ | ✔ |
| 15 | Indole-3-acetyl-L-valine | C_15_H_18_N_2_O_3_ | 2.03 | 275.1416 | 236 | [M+H]^+^ | ✔ | ✔ | X | X |
| 16 | 3,4-Dimethoxyphenethylamine | C_10_H_15_NO_2_ | 2.07 | 182.909 | 164, 104 | M+H | ✔ | ✔ | X | X |
| 17 | Methyl 3-aminopyrazine-2-carboxylic acid | C_6_H_7_N_3_O_2_ | 2.08 | 154.0606 | 155,94 | [M+H]^+^ | ✔ | ✔ | ✔ | ✔ |
| 18 | Tryptamine | C_10_H_12_N_2_ | 2.08 | 161.1213 | 144, 117, 127 | [M+H]^+^ | ✔ | ✔ | ✔ | ✔ |
| 19 | Isoleucylvaline | C_11_H_22_N_2_O_3_ | 2.77 | 231.1508 | 145,213 | [M+H]^+^ | X | ✔ | ✔ | ✔ |
| 20 | 1-methoxyphenazine | C_13_H_10_N_2_O | 3.05 | 210.0926 | 182, 144 | M+H | ✔ | ✔ | X | X |
| 21 | L-Glutamate | C_5_H_9_NO_4_ | 3.09 | 148.0698 | 57,85 | [M+H]^+^ | ✔ | ✔ | ✔ | ✔ |
| 22 | L-Histidine | C_6_H_9_N_3_O_2_ | 3.12 | 156.0708 | 83, 110 | [M+H]^+^ | X | X | ✔ | ✔ |
| 23 | L-tyrosine | C_9_H_11_NO_3_ | 3.14 | 182.0817 | 123,136 | [M+H]^+^ | ✔ | ✔ | ✔ | ✔ |
| 24 | Deoxycarnite | C_7_H_15_NO_2_ | 3.18 | 146.1308 | 60,87 | [M+H]^+^ | ✔ | ✔ | ✔ | ✔ |
| 25 | Indole-3-acetyl-L-leucine | C_16_H_20_N_2_O_3_ | 3.36 | 289.1587 | 130, 243, 271 | [M+H]^+^ | ✔ | ✔ | ✔ | ✔ |
| 26 | Indole-3-ethanol | C_10_H_11_NO | 4.47 | 163.1021 | 147 | M+H | ✔ | ✔ | X | X |
| 27 | Methoxytyramine | C_9_H_13_NO_2_ | 4.74 | 168.0766 | 151,99 | [M+H]^+^ | ✔ | ✔ | X | X |
| 28 | L-Phenylalanine | C_9_H_11_NO_2_ | 4.76 | 166.0872 | 120,121,103 | [M+H]^+^ | ✔ | ✔ | ✔ | ✔ |
| 29 | Cyclo-prolylglycine | C_7_H_10_N_2_O_2_ | 4.86 | 155.0957 | 99 | [M+H]^+^ | ✔ | ✔ | ✔ | ✔ |
| 30 | 3-Ethylpentadecan-2-one | C_17_H_34_O | 5.27 | 254.2045 |  | [M+H]^+^ | ✔ | ✔ | ✔ | ✔ |
| 31 | Cyclo(L-Leu-L-Pro) | C_11_H_18_N_2_O_2_ | 5.33 | 211.119 | 165, 183 | M+H | ✔ | ✔ | ✔ | ✔ |
| 32 | Bacilysocin | C_21_H_43_O_9_P | 5.82 | 471.9185 | 331, 300 | [M+H]^+^ | X | X | ✔ | ✔ |
| 33 | Pyridoxamine | C_8_H_12_N_2_O_2_ | 5.97 | 169.112 | 152 | [M+H]^+^ | ✔ | ✔ | X | X |
| 34 | 7-O-succinyl macrolactin F | C_28_H_38_O_8_ | 6.00 | 488.2423 | 423, 407, 365 | [M+H]^+^ | X | X | ✔ | ✔ |
| 35 | N6-Acetyl-L-lysine | C_8_H_16_N_2_O_3_ | 6.017 | 189.125 | 84,227 | [M+H]^+^ | ✔ | ✔ | ✔ | ✔ |
| 36 | 2-imino-3-(indol-3-yl)propanoic acid | C_19_H_22_N_2_O | 6.37 | 221.0768 | 265 | [M+H]^+^ | X | X | ✔ | ✔ |
| 37 | 1h-indole-3-butanoic acid | C_12_H_13_NO_2_ | 6.49 | 204.1019 | 186 | [M+H]^+^ |  |  |  |  |
| 38 | Ectoine | C_6_H_10_N_2_O_2_ | 6.78 | 143.0849 | 102, 97 | [M+H]^+^ | ✔ | ✔ | X | X |
| 39 | Macrolactin A | C_24_H_34_O_5_ | 7.05 | 402.2021 | 402, 344, 332 | [M+H]^+^ | ✔ | ✔ | ✔ | ✔ |
| 40 | Cyclo(L-Pro-N-ethyl-L-Leu) | C_17_H_34_N_2_O_2_ | 7.83 | 241.1699 |  | [M+H]^+^ | ✔ | ✔ | ✔ | ✔ |
| 41 | Basiliskamide A | C_23_H_31_NO_4_ | 7.99 | 385.2787 | 217 | [M+H]^+^ | X | X | ✔ | X |
| 42 | Phosphoenolpyruvic acid | C_3_H_5_O_6_P | 8.61 | 169.0918 | 86,128 | [M+H]^+^ | ✔ | ✔ | ✔ | ✔ |
| 43 | 7-O-Succinyl macrolactin A | C_28_H_38_O_8_ | 8.63 | 524.3057 | 385, 367, 349 | [M+Na]^+^ | ✔ | ✔ | ✔ | ✔ |
| 44 | N-Methyllysine | C_7_H_16_N_2_O_2_ | 8.89 | 161.1213 | 99, 144 | [M+H]^+^ | ✔ | ✔ | X | X |
| 45 | 1-hexacosanol | C_26_H_54_O | 8.97 | 385.2403 | 303.1769 | [M+H]^+^ | X | ✔ | X | X |
| 46 | 7-O-malonyl macrolactin A | C_27_H_36_O_8_ | 9.44 | 488.3891 |  | [M+H]^+^ | ✔ | ✔ | ✔ | ✔ |
| 47 | N-Methylisoleucine | C_7_H_15_NO_2_ | 9.58 | 168.0766 | 108,130,153 | [M+Na]^+^ | ✔ | ✔ | ✔ | ✔ |
| 48 | Bacillaene | C_34_H_48_N_2_O_6_ | 10.36 | 582.3041 | 181, 223 | [M+H]^+^ | X | X | ✔ | ✔ |
| 49 | L-Prolyl-L-isoleucine | C_11_H_20_N_2_O_3_ | 10.76 | 229.1524 | 182 | [M+H]^+^ | ✔ | ✔ | ✔ | ✔ |
| 50 | 2-heptadecanone | C_17_H_34_O | 11.91 | 255.2714 | 153 | [M+H]^+^ | ✔ | ✔ | ✔ | X |
| 51 | hyocholic acid | C_24_H_40_O_5_ | 12.77 | 158.1679 | 99 | [M+H]^+^ | X | ✔ | X | X |
| 52 | Surfactin A (C14) | C_51_H_89_N_7_O_13_ | 13.07 | 1008.67020 | 227, 557 | [M+H]^+^ | ✔ | ✔ | ✔ | ✔ |
| 53 | Ascorbic acid | C_6_H_8_O_6_ | 13.14 | 177.0672 | 158 | [M+H]^+^ | X | X | ✔ | ✔ |
| 54 | Lichenysin-G6a | C_53_H_94_N_8_O_12_ | 13.56 | 1034.62 |  | [M+H]^+^ | ✔ | ✔ | X | X |
| 55 | Surfactin C (C15) | C_53_H_93_N_7_O_13_ | 14.34 | 1036.733 | 686, 440,544,924, 382 | [M+H]^+^ | ✔ | ✔ | ✔ | ✔ |
| 56 | L-Tryptophan | C_11_H_12_N_2_O_2_ | 15.25 | 205.1019 | 149,99 | [M+H]^+^ | ✔ | ✔ | ✔ | ✔ |
| 57 | Surfactin B | C_52_H_91_N_7_O_13_ | 15.83 | 1045.663 | 1022, 556 | [M+H]^+^ | ✔ | ✔ | X | X |


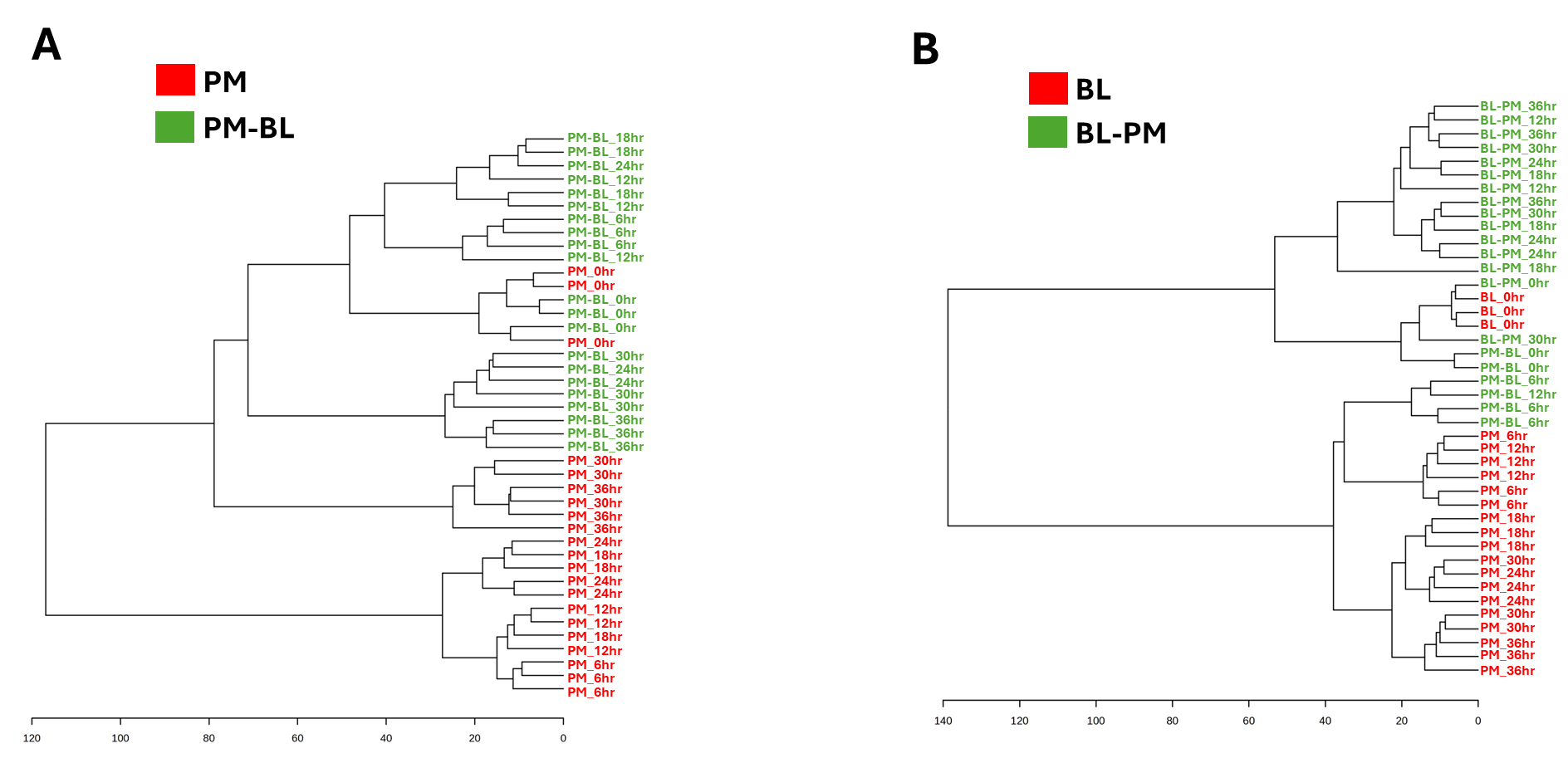


**Figure S1: Comparison of metabolites in control and treated conditions using a dendrogram.** The dendrogram (**A) *P. megaterium* and (B) *B. licheniformis*** represents hierarchical clustering of samples based on their metabolite profiles, highlighting similarities and differences between control and treated groups.
